# Association of Screening and Brief Intervention With Substance Use in Massachusetts Middle and High Schools

**DOI:** 10.1001/jamanetworkopen.2022.26886

**Published:** 2022-08-16

**Authors:** Sharon Levy, Lauren E. Wisk, Machiko Minegishi, Benjamin Ertman, Julie Lunstead, Melissa Brogna, Elissa R. Weitzman

**Affiliations:** 1 Adolescent Substance Use and Addiction Program, Boston Children’s Hospital, Boston, Massachusetts; 2 Division of Developmental Medicine, Boston Children’s Hospital, Boston, Massachusetts; 3 Department of Pediatrics, Harvard Medical School, Boston, Massachusetts; 4 Division of General Internal Medicine and Health Services Research, David Geffen School of Medicine, University of California, Los Angeles, Los Angeles; 5 Division of Adolescent and Young Adult Medicine, Boston Children’s Hospital, Boston, Massachusetts; 6 Computational Health Informatics Program, Boston Children’s Hospital, Boston, Massachusetts

## Abstract

**Importance:**

Screening and brief intervention (SBI) programs in schools have the potential to provide substance use prevention messages to large numbers of adolescents. This study evaluated the association between exposure to a school-based SBI program and reductions in substance use among youths after enactment of a law that required Massachusetts schools to provide SBI to all students.

**Objective:**

To estimate the association between exposure to a school-based SBI program and changes in substance use among youths.

**Design, Setting, and Participants:**

In this mixed-method quality improvement study using an effectiveness-implementation hybrid design, stakeholder interviews were conducted to describe the operations, timing, and impressions of SBI implementation at 14 intervention schools in Massachusetts. Repeated cross-sectional surveys of youths in intervention and comparison groups were administered between December 19, 2017, and May 22, 2019, to assess substance use and associated measures of perceived risk, knowledge, and adult support before and approximately 3 months after SBI implementation among exposed groups. A difference-in-differences framework was used to estimate substance use outcomes associated with SBI exposure among students in middle school (grades 7 and 8) and high school (grades 9 and 10) using adjusted overlap-weighted generalized models to account for covariate imbalance between exposed and unexposed school grades. In addition, 14 school staff members were interviewed about implementation.

**Exposures:**

Exposure vs nonexposure to a school-based SBI program.

**Main Outcomes and Measures:**

Frequency of alcohol, cannabis, and e-cigarette use (measured in days) and any binge drinking in the past 3 months.

**Results:**

Between December 2017 and May 2019, 8771 survey responses were collected from 4587 students in grades 7 through 10 who were attending one of 23 participating school districts. The median (IQR) age was 13 (13-14) years (range, 12-17 years); 2226 students self-identified as female (48.5%), 2206 (48.1%) as male, and 155 (3.4%) as transgender or preferred not to answer. Overall, 163 students (3.6%) identified their race as Asian, 146 (3.2%) as Black or African American, 2952 (64.4%) as White, and 910 (19.8%) as mixed or other race (including American Indian or Alaska Native and Native Hawaiian or Pacific Islander); 416 students (9.1%) preferred not to answer or were missing data on race. A total of 625 students (13.6%) identified their ethnicity as Hispanic and 3962 (86.4%) as non-Hispanic. Cannabis use increased over time in both the SBI group (middle school: marginal estimated probability, 0.73 [95% CI, 0.21-2.51] at baseline vs 2.01 [95% CI, 0.60-6.70] at follow-up; high school: marginal estimated probability, 2.86 [95% CI, 0.56-14.56] at baseline vs 3.10 [95% CI, 0.57-16.96] at follow-up) and the control group (middle school: marginal estimated probability, 0.24 [95% CI, 0.05-1.03] at baseline vs 3.38 [95% CI, 0.81-14.18] at follow-up; high school: marginal estimated probability, 1.30 [95% CI, 0.27-6.29] at baseline vs 1.72 [95% CI, 0.34-8.66] at follow-up). e-cigarette use also increased over time in both the SBI group (middle school: marginal estimated probability, 0.81 [95% CI, 0.22-3.01] at baseline vs 1.94 [95% CI, 0.53-7.02] at follow-up; high school: marginal estimated probability, 3.82 [95% CI, 0.72-20.42] at baseline vs 3.51 [95% CI, 0.55-22.59] at follow-up) and the control group (middle school: marginal estimated probability, 0.51 [95% CI, 0.12-2.30] at baseline vs 3.40 [95% CI, 0.72-16.08] at follow-up; high school: marginal estimated probability, 2.29 [95% CI, 0.41-12.65] at baseline vs 3.53 [95% CI, 0.62-20.16] at follow-up). Exposure to SBI was associated with a significantly smaller increase in the rate of cannabis use among middle school students (adjusted rate ratio [aRR], 0.19; 95% CI, 0.04-0.86) and significantly smaller increases in the rates of cannabis and e-cigarette use among all female students (cannabis use: aRR, 0.17 [95% CI, 0.03-0.96]; e-cigarette use: aRR, 0.16 [95% CI, 0.03-0.82]) compared with nonexposure. No other significant differences were observed among students in grades 7 and 8, and no differences were found in any comparison between groups in grades 9 and 10.

**Conclusions and Relevance:**

In this quality improvement study, exposure to a school-based SBI program was associated with a significantly smaller increase in the rate of cannabis use among middle school students and significantly smaller increases in the rates of cannabis and e-cigarette use among all female students. These findings suggest that implementation of SBI programs in schools may help to reduce substance use among middle school and female students, and further study of these programs is warranted.

## Key Points

**Question:**

Is exposure to a screening and brief intervention (SBI) program in schools associated with reductions in student substance use?

**Findings:**

In this quality improvement study involving 4587 students in grades 7 through 10 who were exposed to a school-based SBI program in Massachusetts, cannabis and e-cigarette use increased over time in all groups. Exposure to SBI was associated with a significantly smaller increase in the rate of cannabis use among middle school students and significantly smaller increases in the rates of cannabis and e-cigarette use among all female students; however, there was no significant difference in any comparison among all high school students.

**Meaning:**

This study’s findings suggest that school-based SBI programs, which are mandatory in Massachusetts, may help to reduce substance use among middle school and female students.

## Teaser

This quality improvement study assesses the association between exposure to a school-based screening and brief intervention program and changes in substance use among students in grades 7 to 10 in Massachusetts.

## Introduction

Screening and brief intervention (SBI) is a framework for primary and secondary substance use prevention. The American Academy of Pediatrics recommends SBI as a component of primary care for adolescents,^1^ and increasing evidence supports its benefit. For example, incorporation of SBI programs in primary care settings in California was associated with reductions in substance use disorders after 3 years.^2^ A multisession school-based brief intervention implemented in New Mexico reduced the frequency of heavy drinking and illicit drug use.^3^ A computer-based SBI model was associated with reductions in alcohol use among adolescents with a chronic illness and decreases in binge drinking among college students with type 1 diabetes.^4^ Expansion of SBI programs is a logical goal because adolescents have relatively low rates of primary care use.^5^ There has been substantial investment in school-based SBI programs. To our knowledge, a controlled study of the potential benefits of SBI outside a private and confidential medical setting has not been conducted.

In 2016, the Commonwealth of Massachusetts began requiring school districts to offer SBI programs to their students. We used the introduction of this state policy as an opportunity to evaluate school-based SBI. This quality improvement study assessed self-reported student substance use before and 3 to 6 months after SBI implementation, comparing students in grades 7 through 10 who attended schools that offered SBI programs to youths in the same grade with students who attended schools that offered SBI programs to youths in different grades, a strategy that allowed same-grade comparison. This study used a difference-in-differences framework to estimate the association between exposure to an SBI program and substance use outcomes. We hypothesized that youths exposed to SBI (SBI group) would have a smaller increase in substance use rates at follow-up compared with youths who were not exposed to SBI (control group).

## Methods

### Study Design

The study protocol was approved by the institutional review board of Boston Children’s Hospital. Before study initiation, a letter or email explaining the study was sent to all parents; parents could opt out of participation for their children. All students provided assent via an anonymous electronic form before completing both the baseline and follow-up surveys. This study followed the Standards for Quality Improvement Reporting Excellence (SQUIRE) reporting guideline for quality improvement studies.

We used an effectiveness-implementation hybrid framework with mixed methods for the study design; to emulate the balanced comparison of a randomized clinical trial, overlap-weighted propensity scores were used to account for imbalance in school grades. During the 2017-2018 and 2018-2019 academic years, we recruited 33 schools from 25 districts in Massachusetts, engaging 38 school grade cohorts among 296 schools contacted (Figure). Three schools from 2 districts encompassing 4 grades were unavailable for follow-up, leaving 30 distinct schools in 23 districts that administered anonymous electronic surveys to all students in selected grades at 2 time points. Over the 2017-2018 and 2018-2019 study years, we assessed nonoverlapping cohorts from 22 schools within 15 districts and 12 schools within 11 districts, respectively.

**Figure.**
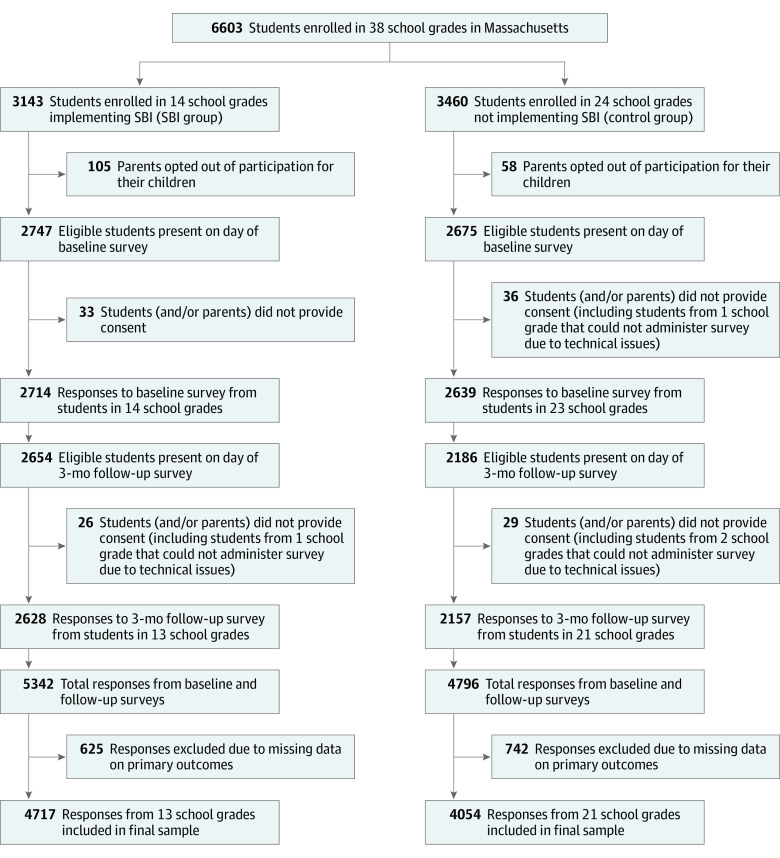
Selection and Exclusion of Participants Massachusetts law and policy dictates the implementation of screening and brief intervention (SBI) programs in schools, and the researchers did not assign them. School grades were included in the SBI group if they received SBI during the 2017-2019 academic years. School grades that could not complete the 3-month follow-up survey were excluded from the analyses. A total of 112 responses to the baseline survey and 114 responses to the 3-month follow-up survey had missing information on school and/or school grade.

### Description of Participating Schools

School data were obtained from the Massachusetts Department of Education website.^6^ Participating school districts ranged in size from 215 to 4986 students, with participating school grades ranging from 36 to 320 students per grade; per-pupil school expenditures varied from $13 000 to $24 000 per annum. One SBI school and 4 control schools were located in rural zip codes. The proportion of students with high need (18.6%-60.9%) and the proportion of students who were economically disadvantaged (5.2%-52.3%) varied.

### SBI Implementation

We conducted 14 phone interviews with school staff members to describe SBI implementation. Interviews lasted approximately 30 to 60 minutes and were recorded and transcribed by study staff (including J.L.).

All schools performed student screening in a private location (conference room, private room in the library, nurse’s office, or school staff member’s office). All but 1 school used a version of the CRAFFT (Car, Relax, Alone, Forget, Friends, Trouble) questionnaire (score range, 0-9, with higher scores indicating greater potential for a substantial problem with substance use)^7^ for screening and an intervention focused on providing brief advice on the risks of alcohol and other drug use (Table 1). Although parents could opt not to have their children participate in the program, the schools all reported high rates (>90%) of student participation (Figure). We collected information on which school grades received screening, how screening was conducted (verbal vs written), the amount of time spent with each student (in minutes), the number of students who received screening at 1 time, and how staff responded to positive or negative screening results.

**Table 1.** Characteristics of Schools Implementing SBI Programs

Characteristic	Schools, No. (%)
Total schools participating in staff interviews, No.	14
School grade	
7	6 (42.9)
8	4 (28.6)
9	3 (21.4)
10	1 (7.1)
Mode of screening administration	
Verbal	11 (78.6)
Combination of verbal and written	3 (21.4)
Time spent per student with a positive screening result, min	
<5	2 (14.3)
5-10	9 (64.3)
>10	3 (21.4)
Time spent per student with a negative screening result, min	
<5	2 (14.3)
5-10	10 (71.4)
>10	2 (14.3)
Screening tool used	
CRAFFT^a^	10 (71.4)
CRAFFT 2.1^b^	3 (21.4)
NIAAA Youth Alcohol Screening Tool^c^	1 (7.1)
Interventions offered for students with a positive screening result	
Brief intervention	9 (64.3)
Resources	2 (14.3)
Internal referral	9 (64.3)
External referral	4 (28.6)
Other (non-SBI)	7 (50.0)
Interventions offered for students with a negative screening result	
Positive reinforcement	11 (78.6)
Brief advice	5 (35.7)
Resources	6 (42.9)
Other	9 (64.3)

Abbreviations: CRAFFT, Car, Relax, Alone, Forget, Friends, Trouble questionnaire; NIAAA, National Institute on Alcoholism and Alcohol Abuse questionnaire; SBI, screening and brief intervention.

^a^ CRAFFT questionnaire^8^ scores range from 0 to 9, with higher scores indicating greater potential for a substantial problem with substance use.

^b^ CRAFFT questionnaire, version 2.1, includes vaping as a method of administration for cannabis use; scores indicate low, medium, or high risk of substance use.

^c^ NIAAA Youth Alcohol Screening Tool scores range from lower risk to high risk, with high risk indicating likelihood of an alcohol use disorder.

### Participants

Before survey administration, a letter or email that explained the study was sent to parents of all 6603 students aged 12 to 17 years in grades 7 to 10 who were enrolled in any of the participating school grades. A total of 163 students (2.5%) were ineligible because their parents opted out of participation (Figure). All other students who were in school on the day the baseline survey was administered (5534 of 6440 students) were eligible for participation and asked to provide assent through an anonymous electronic form advising them of risks and explaining that participation was voluntary. At baseline, 69 students did not consent to participation, 112 survey responses were removed because of missing or incomplete data, and 1 school grade was unable to administer the survey because of technical difficulties. From the remaining 37 school grades, 5353 responses (2714 in the SBI group and 2639 in the control group) to the baseline survey were received.

At follow-up, 4954 students (4840 students in the SBI and control groups and 114 students with no information regarding whether they belonged to the SBI or control group) were eligible to participate and asked to provide assent before taking the survey. In total, 55 students did not consent to participation, 114 survey responses were removed because of missing or incomplete data, and 3 school grades were not able to administer the follow-up survey because of technical difficulties. From the remaining 34 school grades, 4785 responses (2628 in the SBI group and 2157 in the control group) to the follow-up survey were received. Because of missing data on variables of interest (ie, use of alcohol, cannabis, or e-cigarettes and binge alcohol consumption within the past 3 months), 766 of 5353 responses (14.3%) were excluded from the baseline sample, and 601 of 4785 responses (12.6%) were excluded from the follow-up sample, resulting in 1367 of 10 138 total responses (13.5%; 625 from the SBI group and 742 from the control group) excluded from the final analytic sample. The total sample therefore comprised 4587 students (2393 in the SBI group and 2194 in the control group) who submitted 8771 completed surveys (baseline and follow-up combined), with 4717 responses from the SBI group and 4054 responses from the control group (Figure).

### Survey

Data were collected from students via electronic surveys comprising 106 questions at baseline and 114 questions at follow-up; questions were developed based on previous work.^9^ Items included opinions about school screening (7 questions at both baseline and follow-up), getting into trouble in general (7 questions at both baseline and follow-up), alcohol use (8 questions at baseline and 9 questions at follow-up), cannabis use (10 questions at both baseline and follow-up), tobacco and/or e-cigarette use (5 questions at baseline and 4 questions at follow-up), harms associated with substance use (14 questions at baseline and 12 questions at follow-up), attitudes and beliefs (11 questions at both baseline and follow-up), substance use intentions (7 questions at both baseline and follow-up), knowledge about substances (7 questions at baseline and 8 questions at follow-up), social support and general and mental health (10 questions at both baseline and follow-up), demographic characteristics (12 questions at both baseline and follow-up), family history and family attitudes about substance use (4 questions at both baseline and follow-up), future plans (1 question at both baseline and follow-up), and administrative questions (consent: 1 question at both baseline and follow-up; complete confirmation: 1 question at both baseline and follow-up). In addition, 1 free-text question was asked only at baseline, and 10 multiple-choice questions and 1 free-text question about the SBI intervention were asked only at follow-up among SBI groups.

The survey period was December 19, 2017, to May 22, 2019. Surveys were administered anonymously; thus, outcomes were measured at the school grade level. In the SBI group, the baseline survey was administered before SBI screening (Figure), and the follow-up survey was administered approximately 3 months after SBI implementation; in the control group, the surveys were administered approximately 3 months apart. Study data were collected and managed using Research Electronic Data Capture (REDCap)^10,11^ tools hosted at Boston Children’s Hospital (eMethods in the Supplement).

### Outcome Measures

The primary outcome measure was substance use at the school grade level, which was reported on student surveys and measured by the number of days each substance was used and the probability of any binge drinking during the past 3 months. Participants self-reported their frequency of alcohol use (in days) during the past 3 months (“In the past 3 months, on how many days did you have something to drink?”)^12^ and, among those who reported drinking alcohol, their experience of binge drinking in the past 3 months, defined by established age and sex cutoffs.^13^ Participants also reported the frequency of cannabis and e-cigarette use (in days) during the past 3 months (“In the past 3 months, on how many days did you use marijuana/smoke e-cigarettes?”).

Covariates included demographic and health characteristics. Race, ethnicity, and gender were self-reported. Race categories included American Indian or Alaska Native, Asian, Black or African American, Native Hawaiian or Pacific Islander, White, and other race. A category for mixed or other race was created because samples were small; this category included students who selected more than 1 racial category and those who selected American Indian or Alaska Native, Native Hawaiian or Pacific Islander, or other race. Ethnicity categories included Hispanic and non-Hispanic. Gender categories included female, male, and transgender or prefer not to answer. Secondary outcome measures included perceived adult support at school (“If you wanted to talk to someone about a serious problem, do you have an adult in school you could turn to?”), intention to use substances (“In the next 3 months, how likely is it that you will…drink a beverage containing alcohol/use marijuana/smoke e-cigarettes?”), substance use knowledge (assessed by questions pertaining to the negative consequences of substance use among youths, with the knowledge scaled score based on the percentage of questions answered correctly out of 100), and perceived health risk of substance use (“How much is a teenager’s physical or mental health at risk from the following…having 1-2 drinks containing alcohol at least once a month/using marijuana at least once a month/smoking e-cigarettes more than once a month?”) (eMethods in the Supplement).

### Statistical Analysis

Because of the prospective design, we used difference-in-differences estimates to evaluate substance use outcomes among students exposed to SBI compared with students not exposed to SBI, using adjusted and overlap-weighted generalized linear mixed models, assuming an autoregressive covariance structure with random intercepts specified for the school grade levels. Models with a negative binomial distribution were used to assess the changes in substance use frequency during the past 3 months over time, and models with a binomial distribution were used to assess the changes in binge drinking during the past 3 months.

The size and sign of difference-in-differences estimates revealed the extent and direction of change in outcomes among students exposed to SBI, accounting for changes observed among those who were unexposed (eg, substance use was expected to increase naturally with age). Because treatment assignment was not truly random (eg, although all schools implemented an SBI program, administrators could decide which grades would participate, when SBI would be implemented, and how SBI would be administered), estimates were potentially confounded by unobserved differences between the exposed and unexposed groups. Thus, group imbalance was addressed through the use of overlap weighting based on propensity scores, which can achieve covariate balance between exposed and unexposed groups.^14,15,16^ The details of the propensity score calculation are described in eMethods in the Supplement.

Based on previous studies,^17,18,19,20^ a propensity score for SBI assignment was estimated from a multivariable logistic regression model containing per-pupil school expenditures, school grade, proportion of students with high need or economic disadvantages, White race, and female gender. For each level of parental educational status, we dichotomized against all other statuses. Propensity score distributions before and after application of the overlap weights are shown in the eFigure in the Supplement. Covariate differences before and after overlap weighting at the school grade level were assessed using Cohen *d*,^21,22^ which was stratified by school type (eTable 2 and eTable 3 in the Supplement). An absolute Cohen *d* value less than 0.1 was considered negligible. Differences in baseline characteristics between groups were assessed by applying overlap propensity score weights (Table 2).

**Table 2.** Baseline Characteristics After Propensity Score Overlap Weighting by SBI Implemented in Each School Type

Variable	Grades 7 and 8				Grades 9 and 10			
	Unweighted total, No. (%)^a^	Weighted mean probability, %^b^		*P* value	Unweighted total, No. (%)^a^	Weighted mean probability, %^b^		*P* value
		SBI group	Control group			SBI group	Control group	
Total school grades, No.	18	NA	NA	NA	16	NA	NA	NA
Total students, No.	2866	NA	NA	NA	1721	NA	NA	NA
**Student sociodemographic and health characteristics**								
Age, mean (SD), y	12.8 (0.7)	13.0	12.6	.27	14.9 (0.8)	14.7	14.7	.85
Gender^c^								
Female	1388 (48.4)	48.2	51.4	.15	838 (48.7)	55.8	48.1	.049
Male	1380 (48.2)	47.8	45.7	.36	826 (48.0)	40.2	48.9	.03
Transgender or prefer not to answer	98 (3.4)	4.0	2.9	.04	57 (3.3)	4.0	3.0	.17
Race^c^								
Asian	133 (4.6)	5.2	5.1	.97	30 (1.7)	0.8	2.1	.10
Black or African American	94 (3.3)	3.1	2.8	.76	52 (3.0)	1.7	3.0	.16
White	1781 (62.1)	62.5	64.3	.69	1171 (68.0)	75.1	70.1	.13
Mixed or other^d^	602 (21.0)	20.5	19.3	.70	308 (17.9)	16.7	15.5	.53
Missing or prefer not to answer	256 (8.9)	8.7	8.5	.94	160 (9.3)	5.6	9.2	.06
Ethnicity^c^								
Hispanic	391 (13.6)	12.7	10.4	.55	234 (13.6)	8.1	10.0	.47
Non-Hispanic	2475 (86.4)	87.3	89.6	.55	1487 (86.4)	91.9	90.0	.47
Parent educational level of college or higher	2239 (78.1)	77.7	81.5	.29	1155 (67.1)	66.7	70.9	.40
2-Parent household	2274 (79.3)	80.1	80.2	.99	1280 (74.4)	78.9	75.9	.25
Self-rated health								
Good, very good, or excellent	2642 (92.2)	92.1	93.8	.13	1484 (86.2)	89.4	86.6	.20
Fair or poor	176 (6.1)	6.3	4.8	.13	215 (12.5)	9.7	12.5	.22
Missing or prefer not to answer	48 (1.7)	1.6	1.4	.63	22 (1.3)	0.9	0.9	.98
Depression screening (PHQ-2) score ≥3^e^	434 (15.1)	15.0	14.8	.94	440 (25.6)	24.8	26.0	.58
Anxiety health screening (GAD-2) score ≥3^f^	322 (11.2)	10.3	10.8	.82	359 (20.9)	18.5	20.6	.38
**Substance use**								
Alcohol								
Past year	260 (9.1)	9.1	7.8	.46	484 (28.1)	22.7	28.6	.32
Past 3 mo	114 (4.0)	4.2	2.9	.15	332 (19.3)	14.9	20.7	.34
Past 3 mo, mean (SD), d	0.2 (1.4)	0.2	0.2	.48	0.8 (2.7)	0.5	0.8	.17
Any binge drinking in past 3 mo^g^	31 (1.1)	1.3	0.7	.15	168 (9.8)	5.6	10.1	.25
Cannabis								
Past year	93 (3.2)	3.2	2.9	.79	310 (18.0)	12.9	17.9	.22
Past 3 mo	65 (2.3)	2.4	1.9	.63	246 (14.3)	9.7	13.8	.26
Past 3 mo, mean (SD), d	0.3 (3.0)	0.4	0.3	.40	1.7 (7.2)	1.3	1.6	.73
e-Cigarettes								
Past year	137 (4.8)	4.8	4.3	.75	333 (19.3)	13.5	19.9	.21
Past 3 mo	95 (3.3)	3.6	2.7	.43	265 (15.4)	10.1	16.4	.12
Past 3 mo, mean (SD), d	0.4 (4.9)	0.6	0.6	.98	4.1 (16.2)	2.2	4.1	.22
Substance use knowledge, mean (SD), % correct^h^	67.3 (27.0)	67.5	68.8	.48	66.3 (29.6)	68.0	67.7	.87
Perception of substance use as moderate or great health risk^i^								
1-2 Alcohol drinks at least once/mo	1404 (49.0)	50.0	53.2	.58	643 (37.4)	39.5	40.0	.89
Marijuana use at least once/mo	1914 (66.8)	67.8	72.1	.55	828 (48.1)	50.1	51.6	.81
e-Cigarette use more than once/mo	1841 (64.2)	64.3	72.2	.26	929 (54.0)	52.9	56.6	.51
Perception of adult support at school^j^	1721 (60.0)	61.3	60.6	.84	885 (51.4)	52.9	50.5	.45
Definite or probable intention to use substance in next 3 mo^k^								
Alcohol	262 (9.1)	9.3	9.0	.92	384 (22.3)	17.0	22.6	.15
Cannabis	104 (3.6)	3.9	3.5	.76	270 (15.7)	11.9	15.8	.16
e-Cigarettes	96 (3.3)	3.8	2.8	.31	247 (14.4)	9.3	14.9	.23

Abbreviations: GAD-2, 2-item Generalized Anxiety Disorder questionnaire; NA, not applicable; PHQ-2, 2-item Patient Health Questionnaire; SBI, screening and brief intervention.

^a^ The mean or probability of continuous or categorical variables was estimated based on least squares means of fixed effects from the univariate generalized estimating equations with an autoregressive working covariance matrix and Huber-White sandwich estimator, accounting for clustering by school grade.

^b^ After overlap weighting, a single individual no longer represented a single data entry; thus, raw counts were not reported after overlap weighting.

^c^ Gender, race, and ethnicity were self-reported.

^d^ The mixed or other race category was created because the samples were small; this category includes students who selected more than 1 racial category and students who selected American Indian or Alaska Native, Native Hawaiian or Pacific Islander, and/or other race.

^e^ The PHQ-2^23^ uses the first 2 questions of the 9-item PHQ. Scores range from 0 to 6, with higher scores indicating greater likelihood of depression; scores of 3 or greater suggest a major depressive disorder is likely.

^f^ The GAD-2^24^ uses the first 2 questions of the 7-item GAD. Scores range from 0 to 6, with higher scores indicating greater likelihood of generalized anxiety; scores of 3 or greater suggest a generalized anxiety disorder is likely.

^g^ Among those who reported drinking alcohol in the past 3 months and defined by established age and sex cutoffs.^13^

^h^ Assessed by questions pertaining to the negative consequences of substance use among youths, with the knowledge scaled score based on the percentage of questions answered correctly out of 100.

^i^ Perceived riskiness of substance use was assessed by the following Likert-scaled question: “How much is a teenager's physical or mental health at risk from the following…having 1-2 drinks containing alcohol at least once a month/using marijuana at least once a month/smoking e-cigarettes (electronic cigarettes) more than once a month?” The ordinal measure of perceived health risk (with 1 indicating no risk, 2 indicating slight risk, 3 indicating moderate risk, 4 indicating great risk, and 999 indicating prefer not to answer) was then dichotomized for the analysis of responses indicating moderate or great risk vs other responses.

^j^ Perceived adult support at school was assessed by the following question: “If you wanted to talk to someone about a serious problem, do you have an adult in school you could turn to?” The categorical measure of perceived adult support at school (with 1 indicating yes, there is somebody in school I can talk to about a serious problem; 0 indicating no, there is nobody in school I can talk to about a serious problem; and 999 indicating prefer not to answer) was then dichotomized for the analysis of yes responses vs other responses.

^k^ Intention to use a substance during the next 3 months was assessed by the following question: “In the next 3 months, how likely is it that you will…use alcohol/use cannabis/smoke e-cigarettes?” The categorical measure of intent to use a substance (with 1 indicating definitely will not, 2 indicating probably will not, 3 indicating probably will, 4 indicating definitely will, and 999 indicating prefer not to answer) was then dichotomized for the analysis of responses indicating probably or definitely will use a substance vs other responses.

All analyses were stratified by school type (middle school [grades 7 and 8] vs high school [grades 9 and 10]) because we hypothesized that the associations between exposure to SBI and substance use outcomes would differ by school type. Models applied overlapping weights and controlled for age, gender, parental educational level of college or higher, self-rated health (poor, fair, good, very good, excellent, or prefer not to answer), depression screening status (measured by the 2-item Patient Health Questionnaire^23^; score range, 0-6, with higher scores indicating greater likelihood of depression and scores ≥3 suggesting a major depressive disorder is likely; the variable was dichotomized as positive or negative), and anxiety screening status (measured by the 2-item Generalized Anxiety Disorder questionnaire^24^; score range, 0-6, with higher scores indicating greater likelihood of generalized anxiety and scores ≥3 suggesting a generalized anxiety disorder is likely; the variable was dichotomized as positive or negative).

We performed subgroup analyses for female and male students because we hypothesized that the association between SBI exposure and substance use outcomes might differ by gender (other genders, including transgender, were not included due to small sample size)^25,26^ (eTable 8 in the Supplement). We repeated the primary outcome analyses after using overlapping weights only (eTable 6 in the Supplement), after excluding 2 school grades (both grade 9) in which school staff members spent less time with each student, and after stratifying by screening tool used (eTable 9 and eTable 10 in the Supplement).

All statistical analyses were performed using SAS software, version 9.4 (SAS Institute Inc).^27^ The significance threshold was 2-sided *P* = .05.

## Results

### Sample

Between December 19, 2017, and May 22, 2019, 8771 survey responses were collected from 4587 students in grades 7 through 10 who were attending 1 of 23 participating schools (Table 2 and eTable 4 in the Supplement). The median (IQR) age was 13 (13-14) years (range, 12-17 years); 2226 students self-identified as female (48.5%), 2206 (48.1%) as male, and 155 (3.4%) as transgender or preferred not to answer. Overall, 163 students (3.6%) reported their race as Asian, 146 (3.2%) as Black or African American, 2952 (64.4%) as White, and 910 (19.8%) as mixed or other race (including American Indian or Alaska Native and Native Hawaiian or Pacific Islander); 416 students (9.1%) preferred not to answer or were missing data on race. A total of 625 students (13.6%) reported their ethnicity as Hispanic and 3962 (86.4%) as non-Hispanic. At baseline, compared with 315 students excluded from the analysis because of missing data on the primary outcome, students included in the analysis were younger (median [IQR] age, 13 [13-14] years vs 14 [13-15] years; *P* < .001), more likely to be in middle school (2866 students [62.5%] vs 155 students [49.2%]; *P* < .001), and more likely to be female (2226 students [48.5%] vs 136 students [43.2%]; *P* = .02) (eTable 1 in the Supplement). In total, 2781 of 4587 participants (60.6%) agreed or strongly agreed that school staff members should screen students for substance use.

### Middle School Participants

In total, we analyzed 5696 responses (2866 from the baseline survey and 2830 from the follow-up survey) from middle school students (grades 7 and 8). At baseline, the median (IQR) age was 13 (12-13) years; 1388 students (48.4%) self-identified as female, 1380 (48.2%) as male, and 98 (3.4%) as transgender or preferred not to answer. Most participants were White (1781 students [62.1%]), non-Hispanic (2475 students [86.4%]), and came from 2-parent households (2274 students [79.3%]). A total of 434 middle school participants (15.1%) had a positive screening result for depression (Patient Health Questionnaire score ≥3), and 322 (11.2%) had a positive screening result for anxiety (Generalized Anxiety Disorder questionnaire score ≥3). Overall, 2642 participants (92.2%) rated their health as good, very good, or excellent. At baseline, 260 middle school participants (9.1%) reported alcohol use in the past year, 93 (3.2%) reported cannabis use in the past year, and 137 (4.8%) reported e-cigarette use in the past year. After overlap weighting at the school grade level, all baseline demographic and health characteristics in middle school grades (with the exception of age) were balanced between the SBI and control groups. The regression models were adjusted for age. Weighted sample characteristics of middle school participants at the school grade level are shown in Table 2, and unweighted characteristics are shown in eTable 4 and eTable 5 in the Supplement.

### High School Participants

We analyzed 3075 responses (1721 from the baseline survey and 1354 from the follow-up survey) from high school students (grades 9 and 10). At baseline, the median (IQR) age was 15 (14-15) years; 838 participants (48.7%) self-identified as female, 826 (48.0%) as male, and 57 (3.3%) as transgender or preferred not to answer. Most participants were White (1171 students [68.0%]) and non-Hispanic (1487 students [86.4%]) and came from 2-parent households (1280 students [74.4%]). A total of 440 participants (25.6%) had a positive screening result for depression (Patient Health Questionnaire score ≥3), and 359 (20.9%) had a positive screening result for anxiety (Generalized Anxiety Disorder questionnaire score ≥3). Overall, 1484 participants (86.2%) rated their health as good, very good, or excellent. At baseline, 484 high school participants (28.1%) reported alcohol use in the past year, 310 (18.0%) reported cannabis use in the past year, and 333 (19.3%) reported e-cigarette use in the past year. At the school grade level, after weighting, there were no differences observed between the SBI and control groups on any demographic or health variable. Weighted sample characteristics of high school participants at the school grade level are shown in Table 2, and unweighted characteristics are shown in eTable 4 and eTable 5 in the Supplement.

### Substance Use Measures and Perceived Adult Support

Cannabis use increased over time in both the SBI group (middle school students: marginal estimated probability, 0.73 [95% CI, 0.21-2.51] at baseline vs 2.01 [95% CI, 0.60-6.70] at follow-up; high school students: marginal estimated probability, 2.86 [95% CI, 0.56-14.56] at baseline vs 3.10 [95% CI, 0.57-16.96] at follow-up) and the control group (middle school students: marginal estimated probability, 0.24 [95% CI, 0.05-1.03] at baseline vs 3.38 [95% CI, 0.81-14.18] at follow-up; high school students: marginal estimated probability, 1.30 [95% CI, 0.27-6.29] at baseline vs 1.72 [95% CI, 0.34-8.66] at follow-up) (Table 3). e-Cigarette use also increased over time in both the SBI group (middle school students: marginal estimated probability, 0.81 [95% CI, 0.22-3.01] at baseline vs 1.94 [95% CI, 0.53-7.02] at follow-up; high school students: marginal estimated probability, 3.82 [95% CI, 0.72-20.42] at baseline vs 3.51 [95% CI, 0.55-22.59] at follow-up) and the control group (middle school students: marginal estimated probability, 0.51 [95% CI, 0.12-2.30] at baseline vs 3.40 [95% CI, 0.72-16.08] at follow-up; high school students: marginal estimated probability, 2.29 [95% CI, 0.41-12.65] at baseline vs 3.53 [95% CI, 0.62-20.16] at follow-up).

**Table 3.** Differences in Substance Use Changes Between SBI Group vs Control Group From Baseline to Follow-up^a^

Outcome variable	Grades 7 and 8			Grades 9 and 10		
	Marginal estimated probability (95% CI)^b^		aRR (95% CI)^c^	Marginal estimated probability (95% CI)^b^		aRR (95% CI)^c^
	SBI group	Control group		SBI group	Control group	
**Alcohol use in past 3 mo**						
Baseline	0.32 (0.15-0.70)	0.25 (0.10-0.64)	1.02 (0.42-2.47)^d^	1.20 (0.43-3.35)	1.29 (0.48-3.42)	1.79 (0.61-5.30)^d^
Follow-up	0.61 (0.28-1.32)	0.48 (0.20-1.16)		2.50 (0.86-7.27)	1.49 (0.55-4.04)	
**Any binge drinking in past 3 mo**						
Baseline	2.80 (1.10-7.10)	1.40 (0.30-5.50)	0.72 (0.15-3.57)^e^	6.20 (2.10-16.80)	8.50 (3.10-21.70)	1.66 (0.58-4.75)^e^
Follow-up	4.10 (1.70-9.40)	2.70 (0.90-8.50)		7.60 (2.50-20.90)	6.60 (2.20-18.00)	
**Cannabis use in past 3 mo**						
Baseline	0.73 (0.21-2.51)	0.24 (0.05-1.03)	0.19 (0.04-0.86)^f^	2.86 (0.56-14.56)	1.30 (0.27-6.29)	0.82 (0.12-5.65)^f^
Follow-up	2.01 (0.60-6.70)	3.38 (0.81-14.18)		3.10 (0.57-16.96)	1.72 (0.34-8.66)	
**e-Cigarette use in past 3 mo**						
Baseline	0.81 (0.22-3.01)	0.51 (0.12-2.30)	0.36 (0.08-1.59)^g^	3.82 (0.72-20.42)	2.29 (0.41-12.65)	0.60 (0.09-3.78)^g^
Follow-up	1.94 (0.53-7.02)	3.40 (0.72-16.08)		3.51 (0.55-22.59)	3.53 (0.62-20.16)	

Abbreviations: aRR, adjusted rate ratio; SBI, screening and brief intervention.

^a^ All models were overlap weighted and adjusted for age, gender, parental educational level, perceived general health, and depression and anxiety screening results, with 3-way interactions with school types and time point.

^b^ Estimated probability was derived from the models.

^c^ Difference in the change in outcome over time between the SBI vs control groups.

^d^ The aRRs were derived using the difference-in-differences method. Values reflect the difference in the change in the rate of alcohol use (in days) during the past 3 months between the SBI vs control groups from baseline to follow-up.

^e^ Values are estimated adjusted odds ratios comparing the increases in binge drinking during the past 3 months between the SBI vs control groups from baseline to follow-up.

^f^ Difference in the change in the rate of cannabis use (in days) during the past 3 months between the SBI vs control groups from baseline to follow-up.

^g^ Difference in the change in the rate of e-cigarette use (in days) during the past 3 months between the SBI vs control groups from baseline to follow-up.

Among middle school students, the rate of cannabis use at follow-up increased significantly less in the SBI group compared with the control group after adjustment (adjusted rate ratio [aRR], 0.19; 95% CI, 0.04-0.86) (Table 3). No significant differences were observed in alcohol use (aRR, 1.02; 95% CI, 0.42-2.47), binge drinking (adjusted odds ratio [aOR], 0.72; 95% CI, 0.15-3.57), or e-cigarette use (aRR, 0.36; 95% CI, 0.08-1.59) in the past 3 months. Among high school students, no significant differences between the SBI and control groups were found in cannabis use (aRR, 0.82; 95% CI, 0.12-5.65), alcohol use (aRR, 1.79; 95% CI, 0.61-5.30), binge drinking (aOR, 1.66; 95% CI, 0.58-4.75), or e-cigarette use (aRR, 0.60; 95% CI, 0.09-3.78) in the past 3 months.

Among both middle school and high school students, no differences in changes in substance use knowledge (middle school students: β coefficient, −0.13 [95% CI, −4.18 to 3.92]; high school students: β coefficient, −1.22 [95% CI, −7.63 to 5.19]), perceived risk regarding the use of any substance (eg, perception of 1-2 alcohol drinks at least once per month as a moderate or great health risk among middle school students: aOR, 1.19 [95% CI, 0.84-1.68]; among high school students: aOR, 0.95 [95% CI, 0.54-1.66]), or perceived adult support at school (middle school students: aOR, 0.90 [95% CI, 0.63-1.29]; high school students: aOR, 1.09 [95% CI, 0.62-1.92]) were observed between the SBI vs control groups (Table 4; eTable 7 in the Supplement).

**Table 4.** Differences in Changes in Secondary Outcomes Between SBI Group vs Control Group From Baseline to Follow-up

Outcome variable	Marginal estimated probability, % (95% CI)^a^		aOR (95% CI)^b^^,^^c^
	SBI group	Control group	
**Grades 7 and 8** ^d^			
Substance use knowledge, mean (SD), % correct^e^			
Baseline	53.0 (48.9 to 57.1)	53.8 (49.3 to 58.3)	−0.13 (−4.18 to 3.92)^f^
Follow-up	51.9 (47.9 to 55.9)	52.8 (48.3 to 57.3)	
Perception of substance use as moderate or great health risk^g^			
1-2 Alcohol drinks at least once/mo			
Baseline	36.3 (28.4 to 45.0)	35.9 (27.5 to 45.4)	1.19 (0.84 to 1.68)^h^
Follow-up	36.1 (28.4 to 44.5)	31.9 (24.0 to 40.9)	
Marijuana use at least once/mo			
Baseline	49.2 (38.8 to 59.7)	48.1 (36.9 to 59.4)	0.98 (0.66 to 1.44)^h^
Follow-up	46.6 (36.6 to 57.0)	46.1 (35.1 to 57.4)	
e-Cigarette use more than once/mo			
Baseline	50.3 (39.5 to 61.2)	53.9 (42.2 to 65.3)	1.02 (0.69 to 1.49)^h^
Follow-up	49.1 (38.5 to 59.8)	52.3 (40.7 to 63.7)	
Perception of adult support at school^i^			
Baseline	38.4 (30.9 to 46.5)	35.7 (27.7 to 44.6)	0.90 (0.63 to 1.29)^j^
Follow-up	36.6 (29.5 to 44.3)	36.3 (28.3 to 45.2)	
Definite or probable intention to use substance in next 3 mo^k^			
Alcohol			
Baseline	13.3 (8.6 to 20.0)	12.8 (7.8 to 20.4)	0.93 (0.52 to 1.66)^l^
Follow-up	13.3 (8.7 to 19.8)	13.7 (8.5 to 21.5)	
Cannabis			
Baseline	6.1 (3.5 to 10.5)	6.3 (3.2 to 12.2)	1.03 (0.43 to 2.51)^l^
Follow-up	6.9 (4.1 to 11.4)	6.9 (3.6 to 12.9)	
e-Cigarettes			
Baseline	5.9 (2.8 to 12.1)	4.2 (1.7 to 10.0)	0.82 (0.33 to 2.02)^l^
Follow-up	7.6 (3.7 to 14.8)	6.5 (2.8 to 14.1)	
**Grades 9 and 10** ^d^			
Substance use knowledge, mean (SD), % correct^e^			
Baseline	51.1 (45.6 to 56.6)	53.0 (47.7 to 58.3)	−1.22 (−7.63 to 5.19)^f^
Follow-up	52.8 (46.9 to 58.6)	55.9 (50.4 to 61.4)	
Perception of substance use as moderate or great health risk^g^			
1-2 Alcohol drinks at least once/mo			
Baseline	29.0 (20.2 to 39.7)	31.9 (22.8 to 42.7)	0.95 (0.54 to 1.66)^h^
Follow-up	26.8 (18.0 to 38.0)	30.7 (21.5 to 41.7)	
Marijuana use at least once/mo			
Baseline	36.0 (24.6 to 49.2)	38.9 (27.3 to 51.9)	1.04 (0.60 to 1.82)^h^
Follow-up	32.4 (21.2 to 46.1)	34.3 (23.2 to 47.4)	
e-Cigarette use more than once/mo			
Baseline	38.6 (26.4 to 52.5)	45.6 (32.6 to 59.2)	0.91 (0.53 to 1.59)^h^
Follow-up	36.2 (23.9 to 50.7)	45.3 (32.0 to 59.3)	
Perception of adult support at school^i^			
Baseline	32.9 (23.9 to 43.4)	32.4 (23.9 to 42.3)	1.09 (0.62 to 1.92)^j^
Follow-up	36.2 (26.0 to 47.9)	33.7 (24.5 to 44.2)	
Definite or probable intention to use substance in next 3 mo^k^			
Alcohol			
Baseline	15.0 (8.6 to 24.9)	18.3 (11.0 to 28.9)	1.15 (0.59 to 2.25)^l^
Follow-up	18.6 (10.6 to 30.5)	20.1 (12.0 to 31.7)	
Cannabis			
Baseline	10.5 (5.5 to 19.1)	12.7 (7.0 to 22.0)	0.77 (0.34 to 1.73)^l^
Follow-up	9.3 (4.5 to 18.2)	14.3 (7.8 to 24.8)	
e-Cigarettes			
Baseline	8.5 (3.4 to 19.6)	9.8 (4.0 to 22.0)	1.53 (0.65 to 3.59)^l^
Follow-up	12.3 (5.0 to 27.4)	9.7 (3.9 to 22.3)	

Abbreviations: aOR, adjusted odds ratio; SBI, screening and brief intervention.

^a^ Estimated probability was derived from the models.

^b^ All models were adjusted for age, gender, parental educational level, perceived general health, and depression and anxiety screening results, with 3-way interactions with school types and time point.

^c^ The aORs were derived using the difference-in-differences method. Values represent the difference in the change of the outcome over time between the SBI vs control groups.

^d^ Overlap weighted and adjusted.

^e^ Assessed by questions pertaining to the negative consequences of substance use among youths, with the knowledge scaled score based on the percentage of questions answered correctly out of 100.

^f^ Values are β coefficients reflecting the difference in knowledge changes over time between the SBI vs control groups. The difference in knowledge scaled score changes was derived using generalized linear mixed models assuming an autoregressive covariance structure, with random effects by school grade assuming normal distributions.

^g^ Perceived riskiness of substance use was assessed by the following Likert-scaled question: “How much is a teenager's physical or mental health at risk from the following…having 1-2 drinks containing alcohol at least once a month/using marijuana at least once a month/smoking e-cigarettes (electronic cigarettes) more than once a month?” The ordinal measure of perceived health risk (with 1 indicating no risk, 2 indicating slight risk, 3 indicating moderate risk, 4 indicating great risk, and 999 indicating prefer not to answer) was then dichotomized for the analysis of responses indicating moderate or great risk vs other responses.

^h^ The probability of having higher substance use health risk awareness was modeled using overlap-weighted generalized linear mixed models using a logit link and binomial distributions, assuming an autoregressive covariance structure. The estimated aORs compare the increases in having higher health risk awareness between the SBI vs control groups from baseline to follow-up.

^i^ Perceived adult support at school was assessed by the following question: “If you wanted to talk to someone about a serious problem, do you have an adult in school you could turn to?” The categorical measure of perceived adult support at school (with 1 indicating yes, there is somebody in school I can talk to about a serious problem; 0 indicating no, there is nobody in school I can talk to about a serious problem; and 999 indicating prefer not to answer) was then dichotomized for the analysis of yes responses vs other responses.

^j^ The probability of having adult support at school was modeled using overlap-weighted generalized linear mixed models using a logit link and binomial distributions, assuming an autoregressive covariance structure. The estimated aORs compare the increases in having adult support at school between the SBI vs control groups from baseline to follow-up.

^k^ Intention to use a substance during the next 3 months was assessed by the following question: “In the next 3 months, how likely is it that you will…use alcohol/use cannabis/smoke e-cigarettes?” The categorical measure of intent to use a substance (with 1 indicating definitely will not, 2 indicating probably will not, 3 indicating probably will, 4 indicating definitely will, and 999 indicating prefer not to answer) was then dichotomized for the analysis of responses indicating probably or definitely will use a substance vs other responses.

^l^ The changes in the probability of intention to use a substance in the next 3 months over time were assessed by overlap-weighted generalized linear mixed models using a logit link and binomial distributions, assuming an autoregressive covariance structure. The intention to use each substance was operationalized as definitely or probably will use the substance vs definitely or probably will not use the substance (reference variable). The estimated aORs compare the increases in intention to use a substance in the next 3 months between the SBI vs control groups from baseline to follow-up.

Among female students, the rates of cannabis and e-cigarette use at follow-up increased significantly less in the SBI group compared with the control group (cannabis use: aRR, 0.17 [95% CI, 0.03-0.96]; e-cigarette use: aRR, 0.16 [95% CI, 0.03-0.82]) (eTable 8 in the Supplement). No difference was observed among male students in the SBI vs control groups (cannabis use: aRR, 1.17 [95% CI, 0.24-5.80]; e-cigarette use: aRR, 2.50 [95% CI, 0.57-11.06]). In addition, no differences in results by screening method or the amount of time school staff members spent with each student were found (eTables 9-11 in the Supplement).

### SBI Implementation

Although school staff members generally viewed their participation in the SBI program as a positive experience, they expressed concerns about lack of adequate space and time spent outside of learning, and they had questions about the honesty of student responses. No adverse events among students were reported.

## Discussion

This quality improvement study found that although substance use generally increased as students got older, middle school students in a school grade exposed to SBI reported smaller increases in the rate of cannabis use compared with students in the control group, and female students exposed to SBI reported smaller increases in both cannabis and e-cigarette use compared with students in the control group.

Previous work has found that school-based SBI programs are potentially beneficial. Surveys of students^9,28^ and school nurses^29^ have documented positive attitudes toward SBI in schools. A study in Canada^30^ found that, in aggregate, the number of students considered to be at risk of a substance use disorder decreased over time among schools implementing an SBI program. Our results were consistent with these findings, revealing a modest preventive association for school-based SBI programs, specifically among middle school students.

In this study, exposure to an SBI program was not significantly associated with changes in alcohol consumption. Attitudes about alcohol are formed early and have been associated with parenting and social factors.^31^ Positive alcohol expectancies (ie, positive perceptions about the outcomes of drinking alcohol) increase by early adolescence.^32^ Thus, attitudes toward alcohol consumption may be well formed and difficult to alter, even by middle school. It is possible that an intervention with more in-depth content about alcohol use may have greater benefit; future studies could explore this approach. In contrast, opinions about cannabis and e-cigarettes are generally less well formed during adolescence, and clinicians and families are often unsure about how to discuss e-cigarette use with youths.^33,34^ This uncertainty leaves a gap that could be addressed through a brief intervention delivered by a school professional. Our study results suggested that, by high school, attitudes toward cannabis and e-cigarette use are more difficult to alter.

The present study found a significant association between exposure to SBI and changes in the rates of cannabis and e-cigarette use among female students, whereas no difference was observed among male students. Gender-based differences remain an underexplored area in substance use prevention and treatment,^35^ although certain treatment and preventive interventions appear to benefit female individuals more than male individuals.^36^ A future study might explore reasons for substance use and abstention among male students in an effort to identify more beneficial themes for this group. This issue is particularly important because, among high school students, boys are more likely to use substances than girls.^37^

The proportion of students who initiated substance use decreased steadily from 1975 through 2015.^38^ Among adolescents who initiate substance use, the most common trajectory is increasing frequency, intensity, and number of substances used, and a substantial proportion of adolescents develop serious substance use disorders in young adulthood.^37^ Notably, most individuals with addiction start using substances before age 18 years.^8^ The consequences of addiction continue to present challenges in the US, with overdoses associated with more than 100 000 deaths in 2020.^39^ Interventions that prevent, delay, or decrease substance use among young people, even those with nondisordered substance use, could help to change these trajectories. The current study found that implementation of SBI programs was not associated with harms and may decrease substance use trajectories among middle school students. We detected small differences in substance use through exposure to a single brief state-mandated intervention; benefits may be even larger with an intervention that offers enriched youth-centered content about alcohol separately from cannabis and nicotine and with content that is formatively evaluated with regard to engagement of male as well as female individuals. Although we do not yet know the long-term consequences, school-based SBI appears to be a logical component of a broad-based strategy for addressing the nation’s addiction problem.

### Limitations

This study has limitations. All of our data were obtained from student self-report, which is susceptible to recall and social desirability bias. As we expected, the frequency of substance use increased over time (with smaller increases in the SBI group), suggesting that students were likely forthcoming in their report of substance use behaviors and decreasing the likelihood that these biases substantially altered our findings. To protect privacy, all surveys were anonymous, which prevented tracking of substance use trajectories for individual students. This lack of tracking limited the power of our statistical approach, and it is possible that findings would have been strengthened if a true longitudinal design had been used.

Although there is likely to be an almost complete overlap between the students who responded to surveys collected at baseline and follow-up, we could not account for within-individual clustering; however, we accounted for clustering at the school grade level, which should also account for some individual clustering. We used an intention-to-treat model, and we do not know which students actually received SBI, although schools reported high implementation of SBI among their grade-eligible students. It is possible that conducting SBI in only certain grade levels had consequences among students in nonparticipating grades, which would suggest that the actual changes associated with exposure to school-based SBI programs may be greater than those reported in this study.

## Conclusions

In this quality improvement study, exposure to a school-based SBI program was associated with a significantly smaller increase in the rate of cannabis use among middle school students and significantly smaller increases in the rates of cannabis and e-cigarette use among all female students, with no associated harms identified. Implementation of SBI programs in schools may help to reduce substance use among middle school and female students, and further evaluation of these programs is warranted.

## Notes

Supplement. **eMethods.** Survey, Survey Data Interval, School Grade–Level Summary Data for Propensity Score Estimation, Covariates, and Overview of Secondary Outcome Analyses **eTable 1.** Baseline Characteristics of Students Included vs Excluded in the Analytic Sample **eTable 2.** Baseline Characteristics of 2866 Participants in Grades 7 and 8 Before and After Propensity Score Overlap Weighting **eTable 3.** Baseline Characteristics of 1721 Participants in Grades 9 and 10 Before and After Propensity Score Overlap Weighting **eTable 4.** Unweighted Baseline Characteristics of 4587 Total Participants **eTable 5.** Baseline Characteristics of SBI and Control School Grades Before Propensity Score Overlap Weighting, Stratified by School Type **eTable 6.** Differences in Substance Use Behavior Changes Between SBI and Control School Grades From Baseline to Follow-up, Overlap Weighted Without Adjusting for Additional Covariates **eTable 7.** Differences in Changes in Secondary Outcomes Between SBI and Control School Grades From Baseline to Follow-up, Overlap Weighted Without Adjusting for Additional Covariates **eTable 8.** Differences in Substance Use Behavior Changes Between SBI and Control School Grades From Baseline to Follow-up, by Gender **eTable 9.** Differences in Substance Use Behavior Changes Between SBI and Control School Grades in Grades 9 and 10 From Baseline to Follow-up, Excluding 2 Schools With Less Intensive SBI **eTable 10.** Differences in Substance Use Behavior Changes Between SBI and Control School Grades From Baseline to Follow-up When SBI Groups Received the CRAFFT **eTable 11.** Differences in Substance Use Behavior Changes Between SBI and Control School Grades From Baseline to Follow-up When SBI Groups Received the CRAFFT2 and NIAAA **eFigure.** Mirrored Density Plot of Propensity Scores in Full Baseline School Grades, Including 13 School Grades That Received SBI and 21 Control School Grades, Before and After Overlap Weighting
eReferences
