# Errors in the Conflict of Interest Disclosures Statement

**DOI:** 10.1001/jamanetworkopen.2022.38734

**Published:** 2022-10-07

In the Original Investigation titled “Association of Screening and Brief Intervention With Substance Use in Massachusetts Middle and High Schools,”^1^ published August 16, 2022, the Conflict of Interest Disclosures statement has been updated. The amended statement now reads, “Dr Levy reported serving as an expert witness in litigation against JUUL.” This article has been corrected.^1^
