# Supplementary material for: Association of Screening and Brief Intervention With Substance Use in Massachusetts Middle and High Schools
Source: JAMA Netw Open. 2022 Aug 16;5(8):e2226886. doi: 10.1001/jamanetworkopen.2022.26886 (PMC9382442; doi:10.1001/jamanetworkopen.2022.26886)
Supplement: Supplement. — eMethods. Survey, Survey Data Interval, School Grade–Level Summary Data for Propensity Score Estimation, Covariates, and Overview of Secondary Outcome Analyses eTable 1. Baseline Characteristics of Students Included vs Excluded in the Analytic Sample eTable 2. Baseline Characteristics of 2866 Participants in Grades 7 and 8 Before and After Propensity Score Overlap Weighting eTable 3. Baseline Characteristics of 1721 Participants in Grades 9 and 10 Before and After Propensity Score Overlap Weighting eTable 4. Unweighted Baseline Characteristics of 4587 Total Participants eTable 5. Baseline Characteristics of SBI and Control School Grades Before Propensity Score Overlap Weighting, Stratified by School Type eTable 6. Differences in Substance Use Behavior Changes Between SBI and Control School Grades From Baseline to Follow-up, Overlap Weighted Without Adjusting for Additional Covariates eTable 7. Differences in Changes in Secondary Outcomes Between SBI and Control School Grades From Baseline to Follow-up, Overlap Weighted Without Adjusting for Additional Covariates eTable 8. Differences in Substance Use Behavior Changes Between SBI and Control School Grades From Baseline to Follow-up, by Gender eTable 9. Differences in Substance Use Behavior Changes Between SBI and Control School Grades in Grades 9 and 10 From Baseline to Follow-up, Excluding 2 Schools With Less Intensive SBI eTable 10. Differences in Substance Use Behavior Changes Between SBI and Control School Grades From Baseline to Follow-up When SBI Groups Received the CRAFFT eTable 11. Differences in Substance Use Behavior Changes Between SBI and Control School Grades From Baseline to Follow-up When SBI Groups Received the CRAFFT2 and NIAAA eFigure. Mirrored Density Plot of Propensity Scores in Full Baseline School Grades, Including 13 School Grades That Received SBI and 21 Control School Grades, Before and After Overlap Weighting eReferences [file jamanetwopen-e2226886-s001.pdf]

Supplementary Online Content

Levy S, Wisk LE, Minegishi M, et al. Association of screening and brief intervention with substance use in Masschusetts middle and high schools. *JAMA Netw Open*. 2022;5(8):e2226886. doi:10.1001/jamanetworkopen.2022.26886

eMethods. Survey, Survey Data Interval, School Grade–Level Summary Data for Propensity Score Estimation, Covariates, and Overview of Secondary Outcome Analyses

eTable 1. Baseline Characteristics of Students Included vs Excluded in the Analytic Sample

eTable 2. Baseline Characteristics of 2866 Participants in Grades 7 and 8 Before and After Propensity Score Overlap Weighting

eTable 3. Baseline Characteristics of 1721 Participants in Grades 9 and 10 Before and After Propensity Score Overlap Weighting

eTable 4. Unweighted Baseline Characteristics of 4587 Total Participants

eTable 5. Baseline Characteristics of SBI and Control School Grades Before Propensity Score Overlap Weighting, Stratified by School Type

eTable 6. Differences in Substance Use Behavior Changes Between SBI and Control School Grades From Baseline to Follow-up, Overlap Weighted Without Adjusting for Additional Covariates

eTable 7. Differences in Changes in Secondary Outcomes Between SBI and Control School Grades From Baseline to Follow-up, Overlap Weighted Without Adjusting for Additional Covariates

eTable 8. Differences in Substance Use Behavior Changes Between SBI and Control School Grades From Baseline to Follow-up, by Gender

eTable 9. Differences in Substance Use Behavior Changes Between SBI and Control School Grades in Grades 9 and 10 From Baseline to Follow-up, Excluding 2 Schools With Less Intensive SBI

eTable 10. Differences in Substance Use Behavior Changes Between SBI and Control School Grades From Baseline to Follow-up When SBI Groups Received the CRAFFT

eTable 11. Differences in Substance Use Behavior Changes Between SBI and Control School Grades From Baseline to Follow-up When SBI Groups Received the CRAFFT2 and NIAAA

eFigure. Mirrored Density Plot of Propensity Scores in Full Baseline School Grades, Including 13 School Grades That Received SBI and 21 Control School Grades, Before and After Overlap Weighting

eReferences

This supplementary material has been provided by the authors to give readers additional information about their work.

© 2022 Levy S et al. *JAMA Network Open*.

eMethods. Survey, Survey Data Interval, School Grade–Level Summary Data for Propensity Score Estimation, Covariates, and Overview of Secondary Outcome Analyses

Survey

Massachusetts law requires that schools offer SBI to all students at two time points in middle and high school, though districts are given latitude to determine which grades and when in the school year they will screen students. For this project, administrators selected the participating grades and could choose between SBI (those that received SBI) and non-SBI grades. The exposed arm included all student participants who attended a school that selected an SBI-grade for participation in this project; the control arm was comprised of student participants that attended a school that did not deliver SBI to their grade level.

Survey data interval

The data were administered anonymously; thus, the interval between the baseline survey and the follow-up for a unique individual could not be identified. As such, the interval was measured at the school-grade level, based on the survey administration date. Since some schools administered the survey over a few days to a week apart, we computed the interval based on the baseline date and follow-up date that most participants responded. We also estimated the shortest possible and longest possible interval.

Among 34 school grades that participated in the survey at both the baseline and the follow-up, 21 school grades administered the survey over 1-11 days apart at the baseline and mostly 1-7 days apart at the follow-up. Thus, the interval was defined by the date of each survey that >50% of the participants participated. The shortest possible interval was determined by the interval between the latest date that the school grade administered the survey at the baseline and the earliest date of the follow-up survey. In the experimental schools, the baseline survey was administered prior to screening students for substance use, and the follow up was administered approximately 3 months later (median 104 days, IQR 96-113). For the control schools, we similarly administered baseline and follow up surveys approximately 3 months later (median 91 days, range IQR 80-98). Based on the shortest possible and the longest possible interval, the interval range for the experimental school grades was 76-134 days, and that of the control school grades was 43-134 days.

School grade–level summary data for propensity score estimation

The school-grade level summary data for propensity score estimation was created based on the state Department of education (DOE) data and our survey data, including the proportion of each of demographic categories and the mean of continuous variables for each school-grade. The proportion of pupils with economically disadvantaged pupils, high need, annual mean per-pupil expenditure in thousands were provided by DOE and were only available at the school level. Demographic data are based on the survey data.

To calculate the propensity score, we specified treatment models at the school-grade level (the level at which the intervention was determined) to predict the probability of exposure to SBI. We obtained school district or school-grade level characteristics, including school district enrollment size, the per-pupil school expenditure, the proportion of students with high-need or economic disadvantages from the Department of Education (DOE) for the corresponding years of the study operation (see above). The school-grade level summary data for propensity score estimation was then created, based on DOE data and our survey data, which included the proportion of each of demographic categories and mean of continuous variables.

Covariates

Covariates included the per-pupil school expenditure, the proportion of students with high-need or economic disadvantages obtained from the Department of Education (DOE) for the corresponding years of the study operation; self-reported gender, age, and race/ ethnicity (based on fixed categories), parent educational status,perceived parental attitudes about substance use, perceived family history of alcohol problems, self-rated health about over the past 12 months, and depression or anxiety screening status, determined by Patient Health Questionnaire-2 (PHQ-2)[1] and Generalized Anxiety Disorder 2-item (GAD-2)[2] obtained from the survey data. For each level of parent educational status, we dichotomized against all other statuses.

© 2022 Levy S et al. *JAMA Network Open*.

Overview of secondary outcome analyses

Our secondary outcome measures were the knowledge about harms related to substance use, perception of substance use health risks, intention to use substance next 3 months, and perceived adult support at school. The knowledge about substance use harms was assessed by questions related to the negative impact of substance use on youth, determined as the scaled score of the percentage of the questions answered correctly out of 100. The followings are the questionnaires and the correct answer keys;

- Heavy drinking or drug use can harm the developing brain (True)
- Alcohol or drug use will NOT interfere with the ability to learn (False)
- Teens who drink or use other drugs are MORE likely to have unsafe sex (True)
- Brains of teens are LESS susceptible to addiction than brains of adults (False)
- The leading causes of death among teens are NOT related to alcohol and drug use (False)
- The brain is fully developed by age 18 (False)
- Teens who drink are more likely to have problems with school attendance, school work and school conduct (True)
  The following question was added at the follow-up survey.
- Currently in Massachusetts, anyone age 21+ is allowed to possess or use marijuana (True)

Overlap-weighted generalized linear mixed models assuming autoregressive covariance structure with random intercepts specified for the school-grade levels were used to assess the difference in the changes in the mean knowledge scores over time between the SBI groups and the control groups. All the models adjusted for age, gender, parent college education status, self-rated health, depression and anxiety screening status, with 3 way interactions with time points and school types (middle schools/ high schools).

Perceived riskiness of substance use was assessed by Likert scaled questions including “How much is a teenager's physical or mental health at risk from the following... Having 1-2 drinks containing alcohol at least once a month/ Using marijuana at least once a month/ Smoking e-cigarettes (electronic cigarettes) more than once a month”. The ordinal measure of perceived health risk (1, No risk | 2, Slight risk | 3, Moderate risk | 4, Great risk | 999, Prefer not to answer) was then dichotomized for the analyses Moderate/great risk versus other. We assessed the differences in the changes in having higher health risk perception over time between the two groups. Overlap-weighted generalized linear mixed models assuming autoregressive covariance structure with random intercepts specified for the school-grade levels were used for assessing the difference in the changes in the probability of having the Great/moderate risk about substance use health risks over time between the SBI groups and the control groups.

Intention to use substance use was assessed by Likert scaled questions including “In the NEXT THREE MONTHS, how likely is it that you will… Drink a beverage containing alcohol / Use marijuana/Smoke e-cigarettes (electronic cigarettes)”. The ordinal measure of the intention to use respective substance (1, Definitely will not | 2, Probably will not | 3, Probably will | 4, Definitely will | 999, Prefer not to answer) was then dichotomized for the analyses Definitely / Probably will versus other. We assessed the differences in the changes in having the intention to use alcohol/cannabis/ e-cigarettes in next 3 months from the baseline to the follow-up between the two groups. Overlap-weighted generalized linear mixed models, assuming autoregressive (AR) covariance structure with random intercepts specified for the school-grade levels were used.

Perceived adult support at school was assessed with a question “If you wanted to talk to someone about a serious problem, do you have an adult in school you could turn to?” (Yes/No/999, Prefer not to answer). The response was then dichotomized for the analyses as “Yes” versus “Other (No/prefer not to answer)”. Overlap-weighted generalized linear mixed models assuming autoregressive covariance structure with random intercepts specified for the school-grade levels were used for assessing the difference in the changes in the probability of having perceived adult support at school over time between the SBI groups and the control groups.

eTable 1. Baseline Characteristics of Students Included vs Excluded in the Analytic Sample[a]

© 2022 Levy S et al. *JAMA Network Open*.

eTable 1 shows the sample characteristics comparison between the students excluded from the analytic sample due to missing the questionnaires for the primary outcomes (substance use frequency or any binge alcohol consumption).

		**Exclusion for the analytic sample**		
**Variables**	**Total**	**Excluded**	**Included**	**P Value**
**Total,** No.(%)	**(n = 4902)**	**315 (6.4%)**	**4587 (93.6%)**	
**Sociodemographic and health characteristics** No.(%)				
**Age**				
Median (IQR)	13.0 (13.0 – 15.0)	14.0 (13.0 – 15.0)	13.0 (13.0 – 14.0)	<0.001
**School type**				
High schools	1881 (038.4%)	0160 (050.8%)	1721 (037.5%)	<0.001
Middle schools	3021 (061.6%)	0155 (049.2%)	2866 (062.5%)	
**Intervention groups**				
Control	2352 (048.0%)	0158 (050.2%)	2194 (047.8%)	0.42
SBI	2550 (052.0%)	0157 (049.8%)	2393 (052.2%)	
**Gender**				
Male	2366 (048.3%)	0160 (050.8%)	2206 (048.1%)	0.018
Female	2362 (048.2%)	0136 (043.2%)	2226 (048.5%)	
Transgender/PNTA	0174 (003.5%)	0019 (006.0%)	0155 (003.4%)	
**Race**[b]				
Asian	0165 (03.4%)	0002 (00.6%)	0163 (03.6%)	<0.001
Black	0151 (03.1%)	0005 (01.6%)	0146 (03.2%)	
White	2994 (61.1%)	0042 (13.3%)	2952 (64.4%)	
Mixed/other	0939 (19.2%)	0029 (09.2%)	0910 (19.8%)	
PNTA/Missing	0653 (13.3%)	0237 (75.2%)	0416 (09.1%)	
**Ethnicity** [b]				
Hispanics	0652 (013.3%)	0027 (008.6%)	0625 (013.6%)	0.011
**Household composition**				
Other	1286 (026.2%)	0253 (080.3%)	1033 (022.5%)	<0.001
Two parents	3616 (073.8%)	0062 (019.7%)	3554 (077.5%)	
**Parent education**				
Less than college	1225 (025.0%)	0032 (010.2%)	1193 (026.0%)	<0.001
College or higher	3677 (075.0%)	0283 (089.8%)	3394 (074.0%)	
**Depression health screen**				
Positive (PHQ-2 score ≥ 3)[c]	0898 (018.3%)	0024 (007.6%)	0874 (019.1%)	<0.001
**Anxiety health screen**				
Positive (GAD-2 score ≥ 3)[d]	0702 (014.3%)	0021 (006.7%)	0681 (014.8%)	<0.001
**Self-rated health**				
Excellent/very good/good	4397 (089.7%)	0271 (086.0%)	4126 (089.9%)	0.027
Poor/Fair	0426 (008.7%)	0035 (011.1%)	0391 (008.5%)	0.11
PNTA /missing	0079 (001.6%)	0009 (002.9%)	0070 (001.5%)	0.07

**Substance use**				

© 2022 Levy S et al. *JAMA Network Open*.

**Alcohol**				
Past year use	0816 (016.6%)	0072 (022.9%)	0744 (016.2%)	<0.001
Past 3 months use[e]	0505 (010.7%)	0059 (047.2%)	0446 (009.7%)	<0.001
The number of drinking days past 3 months, Median (IQR)[e]	0.0 (0.0 , 0.0)	0.0 (0.0 , 2.0)	0.0 (0.0 , 0.0)	<0.001
Any binge drink past 3 months[f,g]	0215 (004.6%)	0016 (015.4%)	0199 (4.3%)	<0.001
**Cannabis**				
Past year use	0442 (009.0%)	0039 (012.4%)	0403 (008.8%)	<0.001
Past 3 months use[h]	0341 (007.2%)	0030 (025.6%)	0311 (006.8%)	<0.001
The frequency of cannabis use in days past 3 months, Median (IQR)[h]	0.0 (0.0 , 0.0)	0.0 (0.0 , 1.0)	0.0 (0.0 , 0.0)	<0.001
**E-cigarettes**				
Past year use	0493 (010.1%)	0023 (007.3%)	0470 (010.2%)	<0.001
Past 3 months use[i]	0379 (008.2%)	0019 (032.2%)	0360 (007.8%)	<0.001
The frequency of e-cigarettes use in days past 3 months, Median (IQR)[i]	0.0 (0.0 , 0.0)	0.0 (0.0 , 2.0)	0.0 (0.0 , 0.0)	<0.001

Abbreviations: IQR, Interquartile range; PHQ, Patient Health Questionnaire[1]; GAD, General Anxiety Disorder[2]; PNTA, prefer not to answer.
[a] Survey respondent who missed any of the primary outcomes (frequency of alcohol, cannabis or e-cigarette use, or any binge drink) were excluded from the analytic sample. Tests for comparison between the groups for categorical variables included the Chi-squared test, Fisher's exact test, and tests for continuous variables included t-test and the Kruskal–Wallis test.
[b] Self-reported race and ethnicity. Mixed/other means more than 1 of the racial/ethnic categories was selected, or the race selected included any of American Indian/Alaska Native, Hawaiian/Pacific Islander, or race other.
[c] The PHQ-2 inquires about the frequency of depressed mood and anhedonia over the past two weeks. The levels for each of the questions are (0, Not at all | 1, Several days | 2, More than half the days | 3, Nearly every day | 999, Prefer not to answer). The total score of 3 is a cut-off for the positive screening.[1]
[d] The GAD-2 inquires about the frequency of general anxiety symptoms over the past two weeks. The levels for each of the questions are (0, Not at all | 1, Several days | 2, More than half the days | 3, Nearly every day | 999, Prefer not to answer). The total score of 3 is a cut-off for the positive screening.[2]
[e] Overall denominator is n=4712 due to missing values (n=190); those who missed this outcome were excluded from the analytic sample.
[f] Overall denominator is n=4691 due to missing values (n=211); those who missed this outcome were excluded from the analytic sample.
[g] The criteria for binge drink were defined as follows: females 14-17 years old or males/trans/other genders 12-13 years having 3 drinks containing alcohol on one occasion; females 18 years old or males/other genders 14-15 years old having 4 drinks containing alcohol on one occasion; and
males/other genders 16-18 years old having 5 drinks containing alcohol on one occasion.[3]
[h] Overall denominator is n=4704 due to missing values (n=198); those who missed this outcome were excluded from the analytic sample.
[i] Overall denominator is n=4646 due to missing values (n=256); those who missed this outcome were excluded from the analytic sample.

© 2022 Levy S et al. *JAMA Network Open*.

eTable 2. Baseline Characteristics of 2866 Participants in Grades 7 and 8 Before and After Propensity Score Overlap Weighting

eTable 2 and eTable 3 show the covariate differences before and after overlap weighting at the student's level for middle school students and high school students, assessed using the standardized difference (Cohen d). An absolute value of d less than 0.1 is considered a negligible group imbalance.

	**Crude (unweighted)**[a]				**After overlap weighting (weighted)**[g]		
**Variables**	**Total**	**SBI**	**Control**	**Standardized difference**[f]	**SBI**	**Control**	**Standardized difference**[f]
**Total** No.(%)	2866	1946 (67.9%)	920 (32.1%)				
**Sociodemographic and health characteristics** No.(%) or (%)							
**Age**							
Median (IQR)	13.0 (12.0, 13.0)	13.0 (12.0, 13.0)	13.0 (12.0, 13.0)	0.21	13.0 (13.0,13.0)	12.0 (12.0, 13.0)	0.45
**Gender**							
Male	1380 (48.2%)	0937 (48.2%)	0443 (48.2%)	<0.001	47.8%	45.7%	0.04
Female	1388 (48.4%)	0938 (48.2%)	0450 (48.9%)	0.01	48.2%	51.4%	0.07
Transgender/PNTA	0098 (03.4%)	0071 (03.6%)	0027 (02.9%)	0.04	4.0%	2.9%	0.06
**Race**[b]							
Asian	0133 (04.6%)	0095 (04.9%)	0038 (04.1%)	0.04	5.2%	5.1%	0.003
Black	0094 (03.3%)	0067 (03.4%)	0027 (02.9%)	0.03	3.1%	2.8%	0.02
White	1781 (62.1%)	1191 (61.2%)	0590 (64.1%)	0.06	62.5%	64.3%	0.04
Mixed/other	0602 (21.0%)	0422 (21.7%)	0180 (19.6%)	0.05	20.5%	19.3%	0.03
PNTA/ Missing race	256 (8.9%)	171 (8.8)	85 (9.2)	0.02	8.7%	8.5%	0.01
**Ethnicity**[b]							
Hispanics	0391 (13.6%)	0286 (14.7%)	0105 (11.4%)	0.10	12.7%	10.4%	0.07
Non-Hispanics	2475 (86.4%)	1660 (85.3%)	815 (88.6%)	0.10	87.3%	89.6%	0.07
**Parent education**							
College or higher	2239 (78.1%)	1509 (77.5%)	0730 (79.3%)	0.04	77.7%	81.5%	0.09
**Household composition**							
Two parents	2274 (79.3%)	1555 (79.9%)	0719 (78.2%)	0.04	80.1%	80.2%	0.002
**Depression health screen**							
Positive (PHQ-2 score ≥ 3)[c]	0434 (15.1%)	0284 (14.6%)	0150 (16.3%)	0.05	15.0%	14.8%	0.01
**Anxiety health screen**							
Positive (GAD-2 score ≥ 3)[d]	0322 (11.2%)	0201 (10.3%)	0121 (13.2%)	0.09	10.3%	10.8%	0.02
**Self-rated health**							
Excellent/very good/good	2642 (92.2%)	1788 (91.9%)	0854 (92.8%)	0.04	92.1%	93.8%	0.07
Poor/Fair	0176 (06.1%)	0123 (06.3%)	0053 (05.8%)	0.02	6.3%	4.8%	0.06
PNTA /missing	0048 (01.7%)	0035 (01.8%)	0013 (01.4%)	0.03	1.6%	1.4%	0.02

© 2022 Levy S et al. *JAMA Network Open*.

**Substance use** No.(%) or (%)							
**Alcohol**							
Past year use	0260 (09.1%)	0174 (08.9%)	0086 (09.3%)	0.01	9.1%	7.8%	0.05
Past 3 months use	0114 (04.0%)	0082 (04.2%)	0032 (03.5%)	0.04	4.2%	2.9%	0.07
The number of drinking days past 3 months, Median (IQR)	0.0 (0.0, 0.0)	0.0 (0.0, 0.0)	0.0 (0.0, 0.0)	0.01	0.0 (0.0, 0.0)	0.0 (0.0, 0.0)	0.01
Any binge drink past 3 months[e]	0031 (01.1%)	0022 (01.1%)	0009 (01.0%)	0.02	1.3%	0.7%	0.06
**Cannabis**							
Past year use	0093 (03.2%)	0061 (03.1%)	0032 (03.5%)	0.02	3.2%	2.9%	0.02
Past 3 months use	0065 (02.3%)	0043 (02.2%)	0022 (02.4%)	0.01	2.4%	1.9%	0.03
The frequency of cannabis use in days past 3 months, Median (IQR)	0.0 (0.0, 0.0)	0.0 (0.0, 0.0)	0.0 (0.0, 0.0)	0.01	0.0 (0.0, 0.0)	0.0 (0.0, 0.0)	0.03
**E-cigarettes**							
Past year use	0137 (04.8%)	0089 (04.6%)	0048 (05.2%)	0.03	4.8%	4.3%	0.03
Past 3 months use	0095 (03.3%)	0065 (03.3%)	0030 (03.3%)	0.004	3.6%	2.7%	0.05
The frequency of e-cigarettes use in days past 3 months, Median (IQR)	0.0 (0.0, 0.0)	0.0 (0.0, 0.0)	0.0 (0.0, 0.0)	0.01	0.0 (0.0, 0.0)	0.0 (0.0, 0.0)	0.004
**Substance use knowledge (% correct)**[h]							
Mean (SD)	67.3 (27.0)	67.1 (26.9)	67.8 (27.2)	0.03	67.5 (27.0)	68.8 (27.0)	0.05
**Substance use health risk perception**[i] No.(%) or (%)							
**Having 1-2 drinks containing**							
**alcohol at least once a month health risk**							
Great/moderate risk	1404 (49.0%)	0945 (48.6%)	0459 (49.9%)	0.03	50.0%	53.2%	0.07
**Using "marijuana" at least once a month health risk**							
Great/moderate risk	1914 (66.8%)	1282 (65.9%)	0632 (68.7%)	0.06	67.8%	72.1%	0.09
**E-cigarette use more than once a month health risk**							
Great/moderate risk	1841 (64.2%)	1199 (61.6%)	0642 (69.8%)	0.17	64.3%	72.2%	0.17
**Perceived adult support at school**[j] No.(%) or (%)							
**There is somebody in school I can talk to about a serious problem**							
Yes, there is somebody in school I can talk to about a serious problem	1721 (60.0%)	1182 (60.7%)	0539 (58.6%)	0.04	61.3%	60.6%	0.01
**Intent to use substance next 3 months**[k] No.(%) or (%)							
**Intent to use alcohol next 3 months**							

© 2022 Levy S et al. *JAMA Network Open.*

Definitely/probably will	0262 (09.1%)	0168 (08.6%)	0094 (10.2%)	0.05	9.3%	9.0%	0.01
**Intent to use cannabis next 3 months**							
Definitely/probably will	0104 (03.6%)	0070 (03.6%)	0034 (03.7%)	0.01	3.9%	3.5%	0.02
**Intent to use e-cigarette next 3 months**							
Definitely/probably will	0096 (03.3%)	0068 (03.5%)	0028 (03.0%)	0.03	3.8%	2.8%	0.05

Abbreviations: IQR, Interquartile range; PHQ, Patient Health Questionnaire; GAD, General Anxiety Disorder; PNTA, prefer not to answer.[1,2]

[a] Data are presented as percent unless otherwise specified. Column percentage are shown.

[b] Self-reported race and ethnicity. Mixed/other means more than 1 of the racial/ethnic categories was selected, or the race selected included any of American Indian/Alaska Native, Hawaiian/Pacific Islander, or race other.

[c] The PHQ-2 inquires about the frequency of depressed mood and anhedonia over the past two weeks The levels for each of the questions are (0, Not at all | 1, Several days | 2, More than half the days | 3, Nearly every day | 999, Prefer not to answer). A total score of 3 is a cut-off for the positive screening.[1]

[d] The GAD-2 inquires about the frequency of general anxiety symptoms over the past two weeks. The levels for each of the questions are (0, Not at all | 1, Several days | 2, More than half the days | 3, Nearly every day | 999, Prefer not to answer). A total score of 3 is a cut-off for the positive screening.c

[e] The criteria for binge drink were defined as follows: females 14-17 years old or males/trans/other genders 12-13 years having 3 drinks containing alcohol on one occasion; females 18 years old or males/other genders 14-15 years old having 4 drinks containing alcohol on one occasion; and males/other genders 16-18 years old having 5 drinks containing alcohol on one occasion[3].

[f] The standardized difference (d) compares characteristics for SBI assigned school-grade students with students in the control groups (reference group). An absolute value of d of 0.1 or lower indicates a negligible difference in the mean or in the prevalence of a covariate between groups. Absolute values of SMD are shown.

[g] After overlap weighting, a single individual no longer represents a single data entry, thus raw counts are not reported after overlap weighting.

[h] Knowledge scaled score indicates the percentage of questions answered correctly out of 100.

[i] Perceived riskiness of substance use was assessed by Likert scaled questions including
"How much is a teenager's physical or mental health at risk from the following...
Using marijuana at least once a month/ Having 1-2 drinks containing alcohol at least once a month/ Smoking e-cigarettes (electronic cigarettes)
more than once a month". The ordinal measure of perceived health risk (1, No risk | 2, Slight risk | 3, Moderate risk | 4, Great risk | 999, Prefer not to answer) was then dichotomized for the analyses Moderate/great risk versus other.

[j] Perceived adult support at school is assessed by a question “If you wanted to talk to someone about a serious problem, do you have an adult in school you could turn to?”. The categorical measure of perceived adult support at school (1, Yes, there is somebody in school I can talk to about a serious problem | 0, No, there is nobody in school I can talk to about a serious problem | 999, Prefer not to answer) was then dichotomized for the analyses Yes, there is somebody in school I can talk to about a serious problem versus other.

[k] Intent to use substance during the next 3 months was assessed by a question "In the NEXT THREE MONTHS, how likely is it that you will… Smoke e-cigarettes (electronic cigarettes)". The categorical measure of intent to use substance (1, Definitely will not | 2, Probably will not | 3, Probably will | 4, Definitely will | 999, Prefer not to answer) was then dichotomized for the analyses Probably /Definitely will versus other.

© 2022 Levy S et al. *JAMA Network Open*.

eTable 3. Baseline Characteristics of 1721 Participants in Grades 9 and 10 Before and After Propensity Score Overlap Weighting

	Crude (unweighted)[a]				After overlap weighting (weighted)[g]		
	Total	SBI	Control	Standardized difference[f]	SBI	Control	Standardized difference[f]
**Variables**							
**Total** No.(%)	1721	447(26.0%)	1274 (74.0%)				
**Sociodemographic and health characteristics** No.(%) or (%)							
**Age**							
Median (IQR)	15.0 (14.0, 15.0)	15.0 (14.0, 15.0)	15.0 (14.0, 16.0)	0.47	15.0 (14.0, 15.0)	15.0 (14.0, 15.0)	0.08
**Gender Total,** No.(%) **or (%)**							
Male	0826 (48.0%)	0182 (40.7%)	0644 (50.5%)	0.20	40.2%	48.9%	0.18
Female	0838 (48.7%)	0247 (55.3%)	0591 (46.4%)	0.18	55.7%	48.0%	0.16
Transgender/PNTA	0057 (03.3%)	0018 (04.0%)	0039 (03.1%)	0.05	4.0%	3.0%	0.05
**Race** [b]							
Asian	0030 (01.7%)	0004 (00.9%)	0026 (02.0%)	0.10	0.8%	2.1%	0.11
Black	0052 (03.0%)	0008 (01.8%)	0044 (03.5%)	0.10	1.7%	3.0%	0.09
White	1171 (68.0%)	0336 (75.2%)	0835 (65.5%)	0.21	75.2%	70.1%	0.11
Mixed/other	0308 (17.9%)	0072 (16.1%)	0236 (18.5%)	0.06	16.7%	15.5%	0.03
PNTA/ Missing race	160 (9.3 %)	27 (6.0%)	133 (10.4%)	0.16	5.6%	9.2%	0.14
**Ethnicity**[b]							
Hispanics	0234 (13.6%)	0036 (08.1%)	0198 (15.5%)	0.23	8.1%	10.0%	0.07
Non-Hispanics	1487 (86.4%)	411 (91.9%)	1076 (84.5%)	0.23	91.9%	90.0%	0.07
**Parent education**							
College or higher	1155 (67.1%)	0305 (68.2%)	0850 (66.7%)	0.03	66.7%	70.9%	0.09
**Household composition**							
Two parents	1280 (74.4%)	0353 (79.0%)	0927 (72.8%)	0.15	79.0%	75.9%	0.07
**Depression health screen**							
Positive (PHQ-2 score ≥ 3)[c]	0440 (25.6%)	0111 (24.8%)	0329 (25.8%)	0.02	24.9%	26.0%	0.03
**Anxiety health screen**							
Positive (GAD-2 score ≥ 3)[d]	0359 (20.9%)	0083 (18.6%)	0276 (21.7%)	0.08	18.5%	20.6%	0.05
**Self-rated health**							
Excellent/very good/good	1484 (86.2%)	0402 (89.9%)	1082 (84.9%)	0.15	89.4%	86.6%	0.09
Poor/Fair	0215 (12.5%)	0041 (09.2%)	0174 (13.7%)	0.14	9.7%	12.5%	0.09
PNTA/missing	0022 (01.3%)	0004 (00.9%)	0018 (01.4%)	0.05	0.9%	0.9%	0.001
**Substance use** No.(%) or (%)							
**Alcohol**							
Past year use	0484 (28.1%)	0108 (24.2%)	0376 (29.5%)	0.12	22.7%	28.6%	0.14

© 2022 Levy S et al. *JAMA Network Open*.

Past 3 months use	0332 (19.3%)	0075 (16.8%)	0257 (20.2%)	0.09	15.0%	20.7%	0.15
The number of drinking days past 3 months, Median (IQR)	0.0 (0.0, 0.0)	0.0 (0.0, 0.0)	0.0 (0.0, 0.0)	0.14	0.0 (0.0, 0.0)	0.0 (0.0, 0.0)	0.14
Any binge drink past 3 months[e]	0168 (09.8%)	0030 (06.7%)	0138 (10.8%)	0.15	5.7%	10.1%	0.17
**Cannabis**							
Past year use	0310 (18.0%)	0056 (12.5%)	0254 (19.9%)	0.20	12.8%	17.9%	0.14
Past 3 months use	0246 (14.3%)	0043 (09.6%)	0203 (15.9%)	0.19	9.7%	13.8%	0.13
The frequency of cannabis use in days past 3 months, Median (IQR)	0.0 (0.0, 0.0)	0.0 (0.0, 0.0)	0.0 (0.0, 0.0)	0.12	0.0 (0.0, 0.0)	0.0 (0.0, 0.0)	0.07
**E-cigarettes**							
Past year use	0333 (19.3%)	0063 (14.1%)	0270 (21.2%)	0.19	13.5%	19.9%	0.17
Past 3 months use	0265 (15.4%)	0046 (10.3%)	0219 (17.2%)	0.20	10.1%	16.4%	0.19
The frequency of e-cigarettes use in days past 3 months, Median (IQR)	0.0 (0.0, 0.0)	0.0 (0.0, 0.0)	0.0 (0.0, 0.0)	0.18	0.0 (0.0, 0.0)	0.0 (0.0, 0.0)	0.15
**Substance use knowledge (% correct)**[h]							
Mean (SD)	66.3 (29.6)	67.8 (28.8)	65.8 (29.8)	0.07	67.7 (29.1)	68.0 (28.4)	0.01
**Substance use health risk perception**[i] **Total,** No.(%) or (%)							
**Having 1-2 drinks containing**							
**alcohol at least once a month health risk**							
Great/moderate risk	0643 (37.4%)	0176 (39.4%)	0467 (36.7%)	0.06	40.0%	39.4%	0.01
**Using "marijuana" at least once a month health risk**							
Great/moderate risk	0828 (48.1%)	0232 (51.9%)	0596 (46.8%)	0.10	51.6%	50.2%	0.03
**E-cigarette use more than once a month health risk**							
Great/moderate risk	0929 (54.0%)	0233 (52.1%)	0696 (54.6%)	0.05	56.5%	52.9%	0.07
**Perceived adult support at school**[j] **Total,** No.(%) or (%)							
**There is somebody in school I can talk to about a serious problem**							

© 2022 Levy S et al. *JAMA Network Open*.

Yes, there is somebody in school I can talk to about a serious problem	0885 (51.4%)	0234 (52.3%)	0651 (51.1%)	0.03	50.5%	52.9%	0.05
**Intent to use substance next 3 months**[k] **Total,** No.(%) or (%)							
**Intent to use alcohol next 3 months**							
Definitely/probably will	0384 (22.3%)	0079 (17.7%)	0305 (23.9%)	0.16	22.6%	16.9%	0.14
**Intent to use cannabis next 3 months**							
Definitely/probably will	0270 (15.7%)	0053 (11.9%)	0217 (17.0%)	0.15	15.8%	11.9%	0.12
**Intent to use e-cigarette next 3 months**							
Definitely/probably will	0247 (14.4%)	0046 (10.3%)	0201 (15.8%)	0.16	14.9%	9.3%	0.17

Abbreviations: IQR, Interquartile range; PHQ, Patient Health Questionnaire; GAD, General Anxiety Disorder; PNTA, prefer not to answer.

[a] Data are presented as percent unless otherwise specified. Column percentage are shown.

[b] Self-reported race and ethnicity. Mixed/other means more than 1 of the racial/ethnic categories was selected, or the race selected included any of American Indian/Alaska Native, Hawaiian/Pacific Islander, or race other.

[c] The PHQ-2 inquires about the frequency of depressed mood and anhedonia over the past two weeks. The levels for each of the questions are (0, Not at all | 1, Several days | 2, More than half the days | 3, Nearly every day | 999, Prefer not to answer). A total score of 3 is a cut-off for the positive screening.[1]

[d] The GAD-2 inquires about the frequency of general anxiety symptoms over the past two weeks. The levels for each of the questions are (0, Not at all | 1, Several days | 2, More than half the days | 3, Nearly every day | 999, Prefer not to answer). A total score of 3 is a cut-off for the positive screening.[2]

[e] The criteria for binge drink were defined as follows: females 14-17 years old or males/trans/other genders 12-13 years having 3 drinks containing alcohol on one occasion; females 18 years old or males/other genders 14-15 years old having 4 drinks containing alcohol on one occasion; and males/other genders 16-18 years old having 5 drinks containing alcohol on one occasion[3].

[f] The standardized difference (d) compares characteristics for SBI assigned school-grade students with students in the control groups (reference group). An absolute value of d of 0.1 or lower indicates a negligible difference in the mean or in the prevalence of a covariate between groups. Absolute values of SMD are shown.

[g] After overlap weighting, a single individual no longer represents a single data entry, thus raw counts are not reported after overlap weighting.

[h] Knowledge scaled score indicates the percentage of questions answered correctly out of 100.

[i] Perceived riskiness of substance use was assessed by Likert scaled questions including
"How much is a teenager's physical or mental health at risk from the following...
Using marijuana at least once a month/ Having 1-2 drinks containing alcohol at least once a month/ Smoking e-cigarettes (electronic cigarettes)
more than once a month". The ordinal measure of perceived health risk (1, No risk | 2, Slight risk | 3, Moderate risk | 4, Great risk | 999, Prefer not to answer) was then dichotomized for the analyses Moderate/great risk versus other.

[j] Perceived adult support at school is assessed by a question "If you wanted to talk to someone about a serious problem, do you have an adult in school you could turn to?". The categorical measure of perceived adult support at school (1, Yes, there is somebody in school I can talk to about a serious problem | 0, No, there is nobody in school I can talk to about a serious problem | 999, Prefer not to answer) was then dichotomized for the analyses Yes, there is somebody in school I can talk to about a serious problem versus other.

[k] Intent to use substance during the next 3 months was assessed by a question "In the NEXT THREE MONTHS, how likely is it that you will… Smoke e-cigarettes (electronic cigarettes)". The categorical measure of intent to use substance (1, Definitely will not | 2, Probably will not | 3, Probably will | 4, Definitely will | 999, Prefer not to answer) was then dichotomized for the analyses Probably /Definitely will versus other.

© 2022 Levy S et al. *JAMA Network Open*.

eTable 4. Unweighted Baseline Characteristics of 4587 Total Participants

	Crude (Unweighted)		
Variables No.(%)[a]	Overall	Middle Schools GR78	High schools GR910
	4587	2866 (62.5%)	1721 (37.5%)
**Sociodemographic and Health Characteristics**			
Age (median (IQR))	13.0 (13.0, 14.0)	13.0 (12.0, 13.0)	15.0 (14.0, 15.0)
**SBI**	2393 (52.2%)	1946 (67.9%)	447 (26.0%)
**Gender**			
Female	2226 (48.5%)	1388 (48.4%)	838 (48.7%)
Male	2206 (48.1%)	1380 (48.2%)	826 (48.0%)
Transgender/PNTA	155 (3.4%)	98 (3.4%)	57 (3.3%)
**Race** [b]			
Asian	163 (3.6%)	133 (4.6%)	30 (1.7%)
Black	146 (3.2%)	94 (3.3%)	52 (3.0%)
White	2952 (64.4%)	1781 (62.1%)	1171 (68.0%)
Mixed/other	910 (19.8%)	602 (21.0%)	308 (17.9%)
PNTA /Missing	416 (9.1%)	256 (8.9%)	160 (9.3%)
**Ethnicity** [b]			
Non-Hispanics	3962 (86.4%)	2475 (86.4%)	1487 (86.4%)
Hispanics	625 (13.6%)	391 (13.6%)	234 (13.6%)
**Parent education**			
College or higher	3394 (74.0%)	2239 (78.1%)	1155 (67.1%)
**Household composition**			
Two parents	3554 (77.5%)	2274 (79.3%)	1280 (74.4%)
Other	1033 (22.5%)	592 (20.7%)	441 (25.6%)
**Self-rated health**			
Excellent/very good/good	4126 (89.9%)	2642 (92.2%)	1484 (86.2%)
Poor/Fair	391 (8.5%)	176 (6.1%)	215 (12.5%)
PNTA /missing	70 (1.5%)	48 (1.7%)	22 (1.3%)
**Depression screen**			
Positive (PHQ-2 score ≥ 3)[c]	874 (19.1%)	434 (15.1%)	440 (25.6%)
**Anxiety health screen**			
Positive (GAD-2 score ≥ 3)[d]	681 (14.8%)	322 (11.2%)	359 (20.9%)
**Substance use characteristics**			
**Alcohol**			
Lifetime use [e]	996 (21.7%)	400 (14.0%)	596 (34.6%)
Past year use	744 (16.2%)	260 (9.1%)	484 (28.1%)
Past 3 months use	446 (9.7%)	114 (4.0%)	332 (19.3%)
Past 3 months use, in days (median (IQR))	0.0 (0.0, 0.0)	0.0 (0.0, 0.0)	0.0 (0.0, 0.0)
Any binge drink past 3 months [f]	199 (4.3%)	31 (1.1%)	168 (9.8%)
**Cannabis**			
Lifetime use [g]	450 (9.8%)	116 (4.0%)	334 (19.4%)
Past year use	403 (8.8%)	93 (3.2%)	310 (18.0%)
Past 3 months use	311 (6.8%)	65 (2.3%)	246 (14.3%)
Past 3 months use, in days (median (IQR))	0.0 (0.0, 0.0)	0. (0.00, 0.0)	0.0 (0.0, 0.0)
**E-cigarettes**			
Lifetime use [h]	516 (11.2%)	152 (5.3%)	364 (21.2%)

© 2022 Levy S et al. *JAMA Network Open*.

Past year use	470 (10.2%)	137 (4.8%)	333 (19.3%)
Past 3 months use	360 (7.8%)	95 (3.3%)	265 (15.4%)
Past 3 months use, in days (median (IQR))	0.0 (0.0, 0.0)	0.0 (0.0, 0.0)	0.0 (0.0, 0.0)
**Cigarettes**			
Lifetime use [i]	179 (3.9%)	49 (1.7%)	130 (7.6%)
**Any lifetime substance use** [j]			
Alcohol/cannabis/e-cigarettes/cigarettes lifetime use	1168 (25.5%)	476 (16.6%)	692 (40.2%)
**SBI acceptance** [k]			
Strongly agree/agree	2781 (60.6%)	1926 (67.2%)	855 (49.7%)
**Substance use health risk perception** [l]			
Having 1-2 drinks containing alcohol at least once a month health risk: Great/moderate risk	2047 (44.6%)	1404 (49.0%)	643 (37.4%)
Using "marijuana" at least once a month health risk: Great/moderate risk	2742 (59.8%)	1914 (66.8%)	828 (48.1%)
E-cigarette use more than once a month health risk: Great/moderate risk	2770 (60.4%)	1841 (64.2%)	929 (54.0%)
**Substance use knowledge (% correct)**[m]			
Mean (SD)	67.0 (28.0)	67.3 (27.0)	66.3 (29.6)
**Perceived adult support at school** [n]			
**There is somebody in school I can talk to about a serious problem**			
Yes, there is somebody in school I can talk to about a serious problem	2606 (56.8%)	1721 (60.0%)	885 (51.4%)
**Intent to use substance next 3 months** [o]			
**Intent to use alcohol next 3 months**			
Definitely/probably will	646 (14.1%)	262 (9.1%)	384 (22.3%)
**Intent to use cannabis next 3 months**			
Definitely/probably will	374 (8.2%)	104 (3.6%)	270 (15.7%)
**Intent to use e-cigarette next 3 months**			
Definitely/probably will	343 (7.5%)	96 (3.3%)	247 (14.4%)

Abbreviations: SBI, Screening and Brief Intervention, IQR, Interquartile range; PNTA, prefer not to Answer; PHQ, Patient Health Questionnaire; GAD, General Anxiety Disorder.

[a] Data are presented as percent unless otherwise specified.

[b] Self-reported race and ethnicity. Mixed/other means more than 1 of the racial/ethnic categories was selected, or the race selected included any of American Indian/Alaska Native, Hawaiian/Pacific Islander, or race other.

[c] Patient Health Questionnaire-2[1].

[d] The 2-item Generalized Anxiety Disorder scale[2].

[e] The lifetime use of alcohol was asked with the following: “The following questions will ask about your use, if any, of alcohol, tobacco, and other drugs. As a reminder, this survey is anonymous and all of your answers will be kept confidential. Your school and your parent(s)/guardian(s) will NOT have access to any of the individual information that you provide. Alcohol Alcohol includes any kind of beer, wine, wine coolers, or hard liquors like vodka, gin, or whiskey. Each can or bottle of beer, glass of wine or wine cooler, shot of liquor, or mixed drink with liquor in it counts as one "drink".
Not including sips from another person's drink or for religious purposes, have you ever in your whole life had a drink containing alcohol?”- The response choices were “1,Yes 0, No, 999Prefer not to answer”. The response was dichotomized as “Yes” vs “Other (no/prefer not to answer/missing response)” for the analyses.

[f] The criteria for binge drink were defined as follows: females 14-17 years old or males/trans/other genders 12-13 years having 3 drinks containing alcohol on one occasion; females 18 years old or males/other genders 14-15 years old having 4 drinks containing alcohol on one occasion; and males/other genders 16-18 years old having 5 drinks containing alcohol on one occasion[3].

[g] Lifetime use of cannabis was asked with the following: “Marijuana The use of marijuana includes smoking, using a vaporizer, eating edibles, and any other methods of consumption.
Have you ever in your whole life used marijuana? (This includes smoking, using a vaporizer, eating edibles, and any other methods of consuming marijuana)”- The response choices were “1,Yes 0, No, 999Prefer not to answer”. The response was dichotomized as “Yes” vs “Other (no/prefer not to answer/missing response)” for the analyses.

© 2022 Levy S et al. *JAMA Network Open*.

[h] Lifetime use of e-cigarette was asked with the following: “Have you ever in your whole life used electronic cigarettes (e-cigarettes/ tobacco vaporizers)?”- The response choices were “1,Yes 0, No, 999Prefer not to answer”. The response was dichotomized as “Yes” vs “Other (no/prefer not to answer/missing response)” for the analyses.

[i] Lifetime use of cigarette was asked with the following: “Have you ever in your whole life smoked cigarettes, cigars, or used tobacco? (not including any use of e-cigarettes)”- The response choices were “1,Yes 0, No, 999Prefer not to answer”. The response was dichotomized as “Yes” vs “Other (no/prefer not to answer/missing response)” for the analyses.

[j] Self-reported lifetime use of any of alcohol, cannabis, e-cigarette or cigarette (responded to any of these lifetime use questions as “Yes”).

[k] SBI acceptance was assessed with the following: “Screening In some schools, trained staff members (usually nurses, guidance counselors, or psychologists) ask all students about whether they use alcohol and drugs and provide educational information. This process is called screening. How much do you agree with the following statements about screening?

School staff should screen students for substance use (alcohol or drugs).—the response choices ranged from 1. Strongly disagree, 2. Disagree, 3. Agree, 4. Strongly agree, and 999. Prefer not to answer.The response was dichotomized as “strongly agree/agree” vs “other (strongly disagree/disagree/prefer not to answer/missing response)” for the analyses.

[l] Perceived riskiness of substance use was assessed by Likert scaled questions including "How much is a teenager's physical or mental health at risk from the following... Using marijuana at least once a month/ Having 1-2 drinks containing alcohol at least once a month/ Smoking e-cigarettes (electronic cigarettes) more than once a month". The ordinal measure of perceived health risk (1, No risk | 2, Slight risk | 3, Moderate risk | 4, Great risk | 999, Prefer not to answer) was then dichotomized for the analyses Moderate/great risk versus other.

[m] Knowledge scaled score indicates the percentage of questions answered correctly out of 100.

[n] Perceived adult support at school is assessed by a question “If you wanted to talk to someone about a serious problem, do you have an adult in school you could turn to?”. The categorical measure of perceived adult support at school (1, Yes, there is somebody in school I can talk to about a serious problem | 0, No, there is nobody in school I can talk to about a serious problem | 999, Prefer not to answer) was then dichotomized for the analyses Yes, there is somebody in school I can talk to about a serious problem versus other.

[o] Intent to use substance during the next 3 months was assessed by a question "In the NEXT THREE MONTHS, how likely is it that you will… Smoke e-cigarettes (electronic cigarettes)". The categorical measure of intent to use substance (1, Definitely will not | 2, Probably will not | 3, Probably will | 4, Definitely will | 999, Prefer not to answer) was then dichotomized for the analyses Probably /Definitely will versus other.

© 2022 Levy S et al. *JAMA Network Open*.

eTable 5. Baseline Characteristics of SBI and Control School Grades Before Propensity Score Overlap Weighting, Stratified by School Type

		Grade 7&8				Grade 9&10		
**Variables**	**Crude**	**Unweighted**			**Crude**	**Unweighted**		
	**Total**[a]	**SBI**	**Control**		**Total**[a]	**SBI**	**Control**	
	No.(%)	**Mean /Prob (%)**	**Mean /Prob (%)**	**P value**	No.(%)	**Mean /Prob (%)**	**Mean /Prob (%)**	**P value**
School/grades	18	8 (44.4%)	10 (55.6%)		16	5 (31.3%)	11 (68.8%)	
Students	2866	1946 (67.9%)	920 (32.1%)		1721	447 (26.0%)	1274 (74.0%)	
**Sociodemographic and Health Characteristics**								
**Age**								
Mean (SD)	12.8 (0.7)	12.9	12.8	0.59	14.9 (0.8)	14.6	15.0	0.18
**Gender**								
Male	1380 (48.2%)	48.1%	48.2%	0.99	826 (48.0%)	40.7%	50.5%	0.011
Female	1388 (48.4%)	48.2%	48.9%	0.79	838 (48.7%)	55.3%	46.4%	0.017
Transgender/PNTA	98 (3.4%)	3.6%	2.9%	0.26	57 (3.3%)	4.0%	3.1%	0.09
**Race**[b]								
White	1781 (62.1%)	61.2%	64.1%	0.58	1171 (68.0%)	75.1%	65.6%	0.006
Black	94 (3.3%)	3.4%	2.9%	0.63	52 (3.0%)	1.8%	3.5%	0.10
Asian	133 (4.6%)	4.9%	4.1%	0.69	30 (1.7%)	0.9%	2.0%	0.09
Mixed/other	602 (21.0%)	21.7%	19.6%	0.57	308 (17.9%)	16.1%	18.5%	0.37
PNTA /Missing	256 (8.9%)	8.8%	9.2%	0.83	160 (9.3%)	6.1%	10.4%	0.008
**Ethnicity**[b]								
Non-Hispanics	2475 (86.4%)	85.3%	88.6%	0.48	1487 (86.4%)	92.0%	84.5%	0.06
Hispanics	391 (13.6%)	14.7%	11.4%	0.48	234 (13.6%)	8.0%	15.5%	0.06
**Parent education**								
College or higher	2239 (78.1%)	77.5%	79.4%	0.57	1155 (67.1%)	68.2%	66.7%	0.76
**Household composition**								
Two parents	2274 (79.3%)	79.9%	78.1%	0.62	1280 (74.4%)	79.0%	72.8%	0.044
**Self-rated health**								
Excellent/very good/good	2642 (92.2%)	91.9%	92.8%	0.41	1484 (86.2%)	89.9%	84.9%	0.001

© 2022 Levy S et al. *JAMA Network Open*.

Poor/Fair	176 (6.1%)	6.3%	5.8%	0.58	215 (12.5%)	9.2%	13.7%	0.003
PNTA /missing	48 (1.7%)	1.8%	1.4%	0.44	22 (1.3%)	0.9%	1.4%	0.49
**Depression screen**								
Positive (PHQ-2 score ≥ 3)[1]	434 (15.1%)	14.6%	16.3%	0.38	440 (25.6%)	24.8%	25.8%	0.62
**Anxiety health screen**								
Positive (GAD-2 score ≥ 3) [2]	322 (11.2%)	10.3%	13.2%	0.16	359 (20.9%)	18.6%	21.7%	0.15
**Substance use**								
**Alcohol**								
Past year use	260 (9.1%)	8.9%	9.3%	0.83	484 (28.1%)	24.2%	29.5%	0.26
Past 3 months use	114 (4.0%)	4.2%	3.5%	0.49	332 (19.3%)	16.8%	20.2%	0.52
Mean days of use in past 3 months (SD)	0.2 (1.4)	0.2	0.1	0.72	0.8 (2.7)	0.6	0.9	0.09
Any binge drink past 3 months[c]	31 (1.1%)	1.1%	1.0%	0.77	168 (9.8%)	6.7%	10.8%	0.24
**Cannabis**								
Past year use	93 (3.2%)	3.1%	3.5%	0.79	310 (18.0%)	12.5%	19.9%	0.015
Past 3 months use	65 (2.3%)	2.2%	2.4%	0.85	246 (14.3%)	9.6%	15.9%	0.027
Mean days of use in past 3 months (SD)	0.3 (3.0)	0.3	0.2	0.8	1.7 (7.2)	1.1	1.9	0.08
**E-cigarettes**								
Past year use	137 (4.8%)	4.6%	5.2%	0.67	333 (19.3%)	14.1%	21.2%	0.08
Past 3 months use	95 (3.3%)	3.3%	3.3%	0.94	265 (15.4%)	10.3%	17.2%	0.021
Mean days of use in past 3 months (SD)	0.4 (4.9)	0.4	0.4	0.84	4.1 (16.2)	2.1	4.8	0.002
**Substance use knowledge (% correct)**[c]								
Mean (SD)	67.3 (27.0)	67.1	67.8	0.72	66.3 (29.6)	67.8	65.8	0.37
**Substance use health risk perception**[d] N(%) or (%)								
**Having 1-2 drinks containing**								
**alcohol at least once a month health risk**								
Great/moderate risk	1404 (49.0%)	48.6%	49.9%	0.79	0643 (37.4%)	39.4%	36.7%	0.40
**Using "marijuana" at least once a month health risk**								
Great/moderate risk	1914 (66.8%)	65.9%	68.7%	0.64	0828 (48.1%)	51.9%	46.8%	0.36
**E-cigarette use more than once a month health risk**								
Great/moderate risk	1841 (64.2%)	61.6%	69.8%	0.17	0929 (54.0%)	52.1%	54.7%	0.55

© 2022 Levy S et al. *JAMA Network Open*.

**Perceived adult support at school**[e] No.(%) or (%)								
**There is somebody in school I can talk to about a serious problem**								
Yes, there is somebody in school I can talk to about a serious problem	1721 (60.0%)	60.8%	58.6%	0.52	0885 (51.4%)	52.3%	51.1%	0.64
**Intent to use substance next 3 months**[f] No.(%) or (%)								
**Intent to use alcohol next 3 months**								
Definitely/probably will	0262 (09.1%)	8.6%	10.2%	0.50	0384 (22.3%)	17.7%	23.9%	0.06
**Intent to use cannabis next 3 months**								
Definitely/probably will	0104 (03.6%)	3.6%	3.7%	0.94	0270 (15.7%)	11.9%	17.0%	0.013
**Intent to use e-cigarette next 3 months**								
Definitely/probably will	0096 (03.3%)	3.5%	3.0%	0.57	0247 (14.4%)	10.3%	15.8%	0.13

Abbreviations: SBI, Screening and Brief Intervention; PNTA, prefer not to Answer; PHQ, Patient Health Questionnaire; GAD, General Anxiety Disorder.[1,2]

[a] Data are presented as percent unless otherwise specified. The mean or probability of continuous or categorical variables was estimated based on least squares means of fixed effects from the univariate generalized estimating equations with autoregressive working covariance matrix and Huber-White sandwich estimator, accounting for clustering by school grade.

[b] Self-reported race and ethnicity. Mixed/other means more than 1 of the racial/ethnic categories was selected, or the race selected included any of American Indian/Alaska Native, Hawaiian/Pacific Islander, or race other.

[c] The criteria for binge drink were defined as follows: females 14-17 years old or males/trans/other genders 12-13 years having 3 drinks containing alcohol on one occasion; females 18 years old or males/other genders 14-15 years old having 4 drinks containing alcohol on one occasion; and males/other genders 16-18 years old having 5 drinks containing alcohol on one occasion[3].

[d] Knowledge scaled score indicates the percentage of questions answered correctly out of 100.

[e] Perceived riskiness of substance use was assessed by Likert scaled questions including "How much is a teenager's physical or mental health at risk from the following... Using marijuana at least once a month/ Having 1-2 drinks containing alcohol at least once a month/ Smoking e-cigarettes (electronic cigarettes) more than once a month". The ordinal measure of perceived health risk (1, No risk | 2, Slight risk | 3, Moderate risk | 4, Great risk | 999, Prefer not to answer) was then dichotomized for the analyses Moderate/great risk versus other.

[f] Perceived adult support at school is assessed by a question "If you wanted to talk to someone about a serious problem, do you have an adult in school you could turn to?". The categorical measure of perceived adult support at school (1, Yes, there is somebody in school I can talk to about a serious problem | 0, No, there is nobody in school I can talk to about a serious problem | 999, Prefer not to answer) was then dichotomized for the analyses Yes, there is somebody in school I can talk to about a serious problem versus other.

[g] Intent to use substance during the next 3 months was assessed by a question "In the NEXT THREE MONTHS, how likely is it that you will… Smoke e-cigarettes (electronic cigarettes)". The categorical measure of intent to use substance (1, Definitely will not | 2, Probably will not | 3, Probably will | 4, Definitely will | 999, Prefer not to answer) was then dichotomized for the analyses Probably /Definitely will versus other.

© 2022 Levy S et al. *JAMA Network Open*.

eTable 6. Differences in Substance Use Behavior Changes Between SBI and Control School Grades From Baseline to Follow-up, Overlap Weighted Without Adjusting for Additional Covariates

	Weighted, unadjusted [b]: Grade 7&8			Weighted, unadjusted[b]: Grade 9&10		
	Marginal Predictive Values[c]		DID [d]	Marginal Predictive Values[c]		DID [d]
**Outcome Variables**	**SBI group**	**Control group**		**SBI group**	**Control group**	
**Drinking days, past 3 months**	**Mean/Prob (95% CI)**	**Mean/Prob (95% CI)**	**ARR(95% CI)[e]**	**Mean/Prob (95% CI)**	**Mean/Prob (95% CI)**	**ARR(95% CI)[e]**
Baseline	0.17 (0.09-0.30)	0.16 (0.08-0.33)		0.49 (0.21-1.15)	0.64 (0.29-1.44)	
Follow up	0.31 (0.17-0.55)	0.17(0.08-0.36)	1.71 (0.69-4.20)	0.96 (0.39-2.33)	0.85 (0.37-1.93)	1.48 (0.42-5.21)
**Any binge drinking past 3 months past 3 months [a]**			**AOR(95% CI)[f]**			**AOR(95% CI)[f]**
Baseline	1.3% (0.5%-3.4%)	0.6% (0.1%-2.4%)		4.9% (1.6%-13.7%)	6.3% (2.2%-16.7%)	
Follow up	2% (0.8%-5.1%)	1.3% (0.4%-4.1%)	0.72 (0.15-3.51)	6.2% (2.1%-17.0%)	5.3% (1.8%-14.5%)	1.55 (0.56-4.34)
**Cannabis use in days, past 3 months**			**ARR(95% CI)[g]**			**ARR(95% CI)[g]**
Baseline	0.28 (0.11-0.71)	0.17 (0.05-0.60)		1.27 (0.32-4.98)	0.94 (0.23-3.87)	
Follow up	0.46 (0.18-1.15)	0.41 (0.13-1.28)	0.68 (0.15-3.12)	1.84 (0.42-8.11)	1.37 (0.32-5.85)	1.00 (0.12-8.30)
**E-cigarettes use in days, past 3 months**			**ARR(95% CI)[h]**			**ARR(95% CI)[h]**
Baseline	0.44 (0.21-0.96)	0.47 (0.17-1.26)		2.18 (0.66-7.18)	2.69 (0.61-11.86)	
Follow up	0.77 (0.36-1.66)	0.59 (0.21-1.62)	1.37 (0.34-5.58)	2.08 (0.56-7.73)	3.91 (0.97-15.78)	0.66 (0.09-5.07)

Abbreviations: Prob, probability; ARR, adjusted rate ratio; AOR, adjusted odds ratio; DID, difference in differences.

[a] The criteria for binge drink were defined as follows: females 14-17 years old or males/trans/other genders 12-13 years having 3 drinks containing alcohol on one occasion; females 18 years old or males/other genders 14-15 years old having 4 drinks containing alcohol on one occasion; and males/other genders 16-18 years old having 5 drinks containing alcohol on one occasion[3].

[b] All models are overlap-weighted and include the 3way interactions with school types and time point.

[c] Predicted probability is derived from the models.

[d] The intervention effect represents the difference in the change of the outcome over time between the SBI vs the control arm.

[e] Difference in the changes in the rate of drinking days during the past 3months between SBI and control groups from the baseline to the follow-up.

[f] The estimated odds ratio comparing the increases from the baseline to follow-up in binge drinking during the past 3 months between the SBI to the control arms.

[g]Difference in the changes in the rate of cannabis use in days during the past 3months between SBI and control groups from the baseline to the follow-up.

[h] Difference in the changes in the rate of e-cigarette use in days during the past 3months between SBI and control groups from the baseline to the follow-up.

© 2022 Levy S et al. *JAMA Network Open.*

eTable 7. Differences in Changes in Secondary Outcomes Between SBI and Control School Grades From Baseline to Follow-up, Overlap Weighted Without Adjusting for Additional Covariates

	Weighted, unadjusted: Grade 7&8			Weighted, unadjusted: Grade 9&10		
	Marginal Predictive Values [d]		DID [e]	Marginal Predictive Values [d]		DID [e]
**Variables**	**SBI group**	**Control group**		**SBI group**	**Control group**	
**Substance use health risk intolerance** [a]	**Prob (95%CI)**	**Prob (95%CI)**		**Prob (95%CI)**	**Prob (95%CI)**	
**Having 1-2 drinks containing alcohol at least once a month health risk**						
**Moderate /great risk**						
Baseline	50.1% (41.9%-58.4%)	50.6% (41.6%-59.5%)	**AOR(95%CI)** [f]	39.2% (29.1%-50.3%)	40.9% (31.0%-51.7%)	**AOR(95%CI)** [f]
Follow up	48.9% (40.7%-57.2%)	45.4% (36.5%-54.5%)	1.17 (0.84-1.65)	36.2% (26.0%-47.8%)	38.9% (28.9%-49.8%)	0.96 (0.56-1.67)
**Using "marijuana" at least once a month health risk**						
**Moderate /great risk**						
Baseline	69.2% (59.3%-77.6%)	69.1% (58.5%-78.0%)	**AOR(95%CI)** [f]	50.5% (36.9%-64.0%)	52.6% (39.2%-65.6%)	**AOR(95%CI)** [f]
Follow up	65.6% (55.3%-74.6%)	66.2% (55.2%-75.7%)	0.97 (0.67-1.41)	46.1% (32.5%-60.4%)	46.9% (33.7%-60.4%)	1.06 (0.62-1.81)
**E-cigarette use more than once a month health risk**						
**Moderate /great risk**						
Baseline	66.4% (56.4%-75.2%)	70.0 % (59.7%-78.6%)	**AOR (95%CI)**[f]	51.6% (38.1%-64.8%)	57.6% (44.3%-69.8%)	**AOR(95%CI)** [f]
Follow up	64.3% (54.0%-73.4%)	67.8% (57.2%-76.9%)	1.01 (0.69-1.46)	48.7% (35.0%-62.5%)	56.5% (43.1%-69.1%)	0.93 (0.54-1.59)
**Substance use knowledge**	**Mean/Prob (95% CI)**	**Mean/Prob (95% CI)**		**Mean/Prob (95% CI)**	**Mean/Prob (95% CI)**	
**(% correct)**[b]						
Baseline	66.8 (62.4-71.1)	68.1 (63.4-72.9)	**Diff (95%CI)** [g]	67.5 (61.6-73.4)	68.2 (62.6-73.9)	**Diff (95%CI)** [g]
Follow up	64.6 (60.2-69.0)	66.7 (61.9-71.6)	-0.7(-5.4, 3.9)	68.8 (62.5-75.0)	70.8 (64.9-76.6)	-1.3 (-8.6, 6.1)
**Perceived adult support at school**						
**There is somebody in school I can talk to about a serious Problem**						
Baseline	61.4% (54.8%-67.6%)	58.9% (51.3%-66.0%)	**AOR (95%CI)** [h]	53.5% (44.0%-62.8%)	51.4% (42.4%-60.3%)	**AOR (95%CI)** [h]
Follow up	58.3% (51.6%-64.8%)	58.5% (50.8%-65.9%)	0.89 (0.63-1.26)	56.9% (46.7%-66.5%)	51.8% (42.5%-61.1%)	1.13 (0.66-1.92)
**Intent to use substance next 3 months** [c]						

© 2022 Levy S et al. *JAMA Network Open*.

**Intent to use alcohol next 3 months**						
Baseline	10.2% (6.5%-15.6%)	9.5% (5.8%-15.3%)	**AOR (95%CI)** [i]	16% (9.3%-26.2%)	19.4% (11.7%-30.3%)	**AOR (95%CI)** [i]
Follow up	10.6% (6.8%-16.1%)	10.6% (6.5%-17.0%)	0.92 (0.52-1.64)	19.9% (11.6%-31.8%)	21.8% (13.3%-33.6%)	1.12 (0.58-2.17)
**Intent to use cannabis next 3 months**						
Baseline	3.7% (2.0%-6.7%)	3.5% (1.7%-7.0%)	**AOR (95%CI)** [i]	11.0% (5.6%-20.3%)	13.0% (7.0%-23.0%)	**AOR (95%CI)** [i]
Follow up	4.4% (2.4%-7.7%)	4.0% (2.0%-7.9%)	1.03 (0.43-2.47)	10.0% (4.9%-19.4%)	15.4% (8.4%-26.6%)	0.74 (0.34-1.64)
**Intent to use e-cigarette next 3 months**						
Baseline	4.0% (1.9%-8.3%)	2.7% (1.1%-6.3%)	**AOR (95%CI)** [i]	8.0% (3.3%-18.3%)	9.1% (3.8%-20.3%)	**AOR (95%CI)** [i]
Follow up	5.2% (2.5%-10.3%)	4.2% (1.8%-9.4%)	0.80 (0.33-1.95)	11.5% (4.8%-24.9%)	9.3% (3.9%-20.9%)	1.44 (0.63-3.33)

Abbreviations: Prob, probability; ARR, adjusted rate ratio; AOR, adjusted odds ratio; Diff, difference; DID, difference in differences; Diff, Difference in beta.

[a] Perceived riskiness of substance use was assessed by Likert scaled questions including "How much is a teenager's physical or mental health at risk from the following... Using marijuana at least once a month/ Having 1-2 drinks containing alcohol at least once a month/ Smoking e-cigarettes (electronic cigarettes) more than once a month". The ordinal measure of perceived health risk (1, No risk | 2, Slight risk | 3, Moderate risk | 4, Great risk | 999, Prefer not to answer) was then dichotomized for the analyses Moderate/great risk versus other.

[b] Substance use knowledge scaled score indicates the percentage of questions answered correctly out of 100.The difference in knowledge score changes was modeled with generalized linear mixed effect models assuming a normal distribution, with random effects by school grade. The beta coefficients were shown as the difference in knowledge changes over time comparing the two arms.

[c] Intent to use substance during the next 3 months was assessed by a question "In the NEXT THREE MONTHS, how likely is it that you will… Smoke e-cigarettes (electronic cigarettes)". The categorical measure of intent to use substance (1, Definitely will not | 2, Probably will not | 3, Probably will | 4, Definitely will | 999, Prefer not to answer) was then dichotomized for the analyses Probably /Definitely will versus other.

[d] Predicted probability is derived from the models.

[e] This represents the difference in the change of the outcome over time between the SBI vs the control arm.

[f] The probability of having the higher substance use health risk awareness (operationalized as perceiving the statement as moderate/great risk vs. Slight risk/no risk/prefer not to answer or missing answers) was modeled using overlap-weighted generalized linear mixed models using a logit link assuming binomial distributions. Intervention effects were described as adjusted odds ratios comparing the two arms over time instead of the beta coefficients.

[g] Differences in the changes in the scaled substance use knowledge (% of the correct answers) over time between the SBI and the control arms.

[h] The estimated odds ratio compares the increases from baseline to follow-up in having adult support at school between the SBI and the control arms.

[i] The estimated odds ratio compares the increases from the baseline to follow-up in intent to use the substance in the next 3 months between the SBI and the control arms.

© 2022 Levy S et al. *JAMA Network Open*.

eTable 8. Differences in Substance Use Behavior Changes Between SBI and Control School Grades From Baseline to Follow-up, by Gender

	weighted, adjusted			weighted, unadjusted		
	Marginal Predictive Values[a]		DID [c,d]	Marginal Predictive Values[a]		DID[d]
**Variables**	**SBI group**	**Control group**		**SBI group**	**Control group**	
**Female gender**	**Mean/Prob (95% CI)**	**Mean/Prob (95% CI)**		**Mean/Prob (95% CI)**	**Mean/Prob (95% CI)**	
**The frequency of drinking days past 3 months**			**ARR (95% CI)[e]**			**ARR(95% CI)[e]**
Baseline	0.20 (0.09-0.46)	0.24 (0.10-0.57)		0.12 (0.04-0.32)	0.15 (0.05-0.44)	
Follow up	0.46 (0.21-1.02)	0.45 (0.19-1.07)	1.19 (0.46-3.13)	0.27 (0.10-0.73)	0.33 (0.11-0.95)	1.08 (0.41-2.89)
**Any binge drinking[b] during the past 3 months**			**AOR (95% CI)[f]**			**AOR(95% CI)[f]**
Baseline	3.4% (1.5%-7.7%)	3.5% (1.4%-8.3%)		2.2% (0.8%-6.1%)	2.2% (0.7%-6.4%)	
Follow up	4.0% (1.8%-8.8%)	3.4% (1.4%-8.2%)	1.2 (0.40-3.61)	2.9% (1.0%-7.7%)	2.3% (0.7%-6.7%)	1.27 (0.43-3.76)
**The frequency of cannabis use in days past 3 months**			**ARR (95% CI)[g]**			**ARR (95% CI)[g]**
Baseline	0.45 (0.16-1.26)	0.21 (0.06-0.67)		0.21 (0.05-0.77)	0.11 (0.03-0.51)	
Follow up	0.56 (0.21-1.53)	1.50 (0.42-5.38)	**0.17 (0.03-0.96)**	0.52 (0.13-2.03)	0.89 (0.18-4.31)	0.32 (0.06-1.88)
**The frequency of E-cigarettes use in days past 3 months**			**ARR (95% CI)[h]**			**ARR(95% CI)[h]**
Baseline	0.63 (0.20-2.02)	0.50 (0.14-1.82)		0.42 (0.12-1.55)	0.34 (0.08-1.40)	
Follow up	0.68 (0.21-2.20)	3.44 (0.80-14.82)	**0.16 (0.03-0.82)**	0.58 (0.16-2.18)	1.67 (0.43-6.48)	0.25 (0.05-1.34)
**Male gender**	**Mean/Prob (95% CI)**	**Mean/Prob (95% CI)**		**Mean/Prob (95% CI)**	**Mean/Prob (95% CI)**	
**The frequency of drinking days past 3 months**			**ARR (95% CI)[e]**			**ARR (95% CI)[e]**
Baseline	0.47 (0.21-1.04)	0.41 (0.17-0.98)		0.24 (0.09-0.65)	0.17 (0.06-0.50)	
Follow up	0.88 (0.40-1.94)	0.44 (0.19-1.05)	1.72 (0.67-4.42)	0.61 (0.22-1.64)	0.27 (0.09-0.78)	1.62 (0.61-4.32)
**Any binge drinking[b] during the past 3 months**			**AOR (95% CI)[f]**			**AOR (95% CI)[f]**
Baseline	2.3% (0.9%-5.9%)	2.8% (1.1%-7.1%)		1.5% (0.5%-4.6%)	1.6% (0.5%-4.8%)	
Follow up	3.7% (1.6%-8.4%)	2.3% (0.8%-6.0%)	2.06 (0.56-7.65)	2.7% (1.0%-7.6%)	1.4% (0.4%-4.5%)	2.09 (0.57-7.65)
**The frequency of cannabis use in days past 3 months**			**ARR (95% CI)[g]**			**ARR (95% CI)[g]**
Baseline	0.59 (0.20-1.74)	0.33 (0.10-11)		0.35 (0.09-1.41)	0.13 (0.03-0.60)	
Follow up	2.03 (0.71-5.83)	0.96 (0.29-3.16)	1.17 (0.24-5.80)	1.29 (0.33-5.14)	0.56 (0.12-2.60)	0.88 (0.16-4.90)
**The frequency of E-cigarettes use in days past 3 months**			**ARR (95% CI)[h]**			**ARR (95% CI)[h]**
Baseline	1.29 (0.38-4.36)	0.75 (0.20-2.79)		0.47 (0.12-1.74)	0.34 (0.08-1.45)	

© 2022 Levy S et al. *JAMA Network Open*.

Follow up	3.65 (1.04-12.76)	0.84 (0.22-3.20)	2.50 (0.57-11.06)	1.67 (0.43-6.48)	0.49 (0.11-2.13)	2.52 (0.52-12.16)

Abbreviations: Prob, probability; ARR, adjusted rate ratio; AOR, adjusted odds ratio; DID, difference in differences. **Bolded where p value<0.05.**

[a] Predicted probability is derived from the models.

[b] The criteria for binge drink were defined as follows: females 14-17 years old or males/trans/other genders 12-13 years having 3 drinks containing alcohol on one occasion; females 18 years old or males/other genders 14-15 years old having 4 drinks containing alcohol on one occasion; and males/other genders 16-18 years old having 5 drinks containing alcohol on one occasion[3].

[c] All models adjust for age, parent education, perceived general health, depression and anxiety screening, with the 3way interactions with gender and time point.

[d] Difference in differences represents the difference in the change of the outcome over time between the SBI versus the control arm.

[e] Difference in the changes in the rate of drinking days during the past 3months between SBI and control groups from the baseline to the follow-up.

[f] The estimated odds ratio compares the increases from the baseline to follow-up in binge drinking during the past 3 months between the SBI and the control arms.

[g] Difference in the changes in the rate of cannabis use in days during the past 3months between SBI and control groups from the baseline to the follow-up.

[h] Difference in the changes in the rate of e-cigarette use in days during the past 3months between SBI and control groups from the baseline to the follow-up.

© 2022 Levy S et al. *JAMA Network Open*.

eTable 9. Differences in Substance Use Behavior Changes Between SBI and Control School Grades in Grades 9 and 10 From Baseline to Follow-up, Excluding 2 Schools With Less Intensive SBI

eTable 9 shows the results for the sensitivity analyses; we repeated the primary outcome analyses after excluding 2 schools (all grade 9, one school participated both years) which reported less than5 minutes of SBI for the students' interview. A total of N=337 grade 9 students 'responses were excluded from the baseline survey and a total of N=320 grade 9 students 'responses were excluded from the follow-up survey. The primary outcome analyses result for grade 9 and 10 are summarized in eTable4.

	**Weighted, adjusted: Grade 9&10**			**Weighted, unadjusted: Grade 9&10**		
	**Marginal Predictive Values[a]**		**DID [c,d]**	**Marginal Predictive Values[a]**		**DID [c,d]**
**Variables**	**SBI group**	**Control group**		**SBI group**	**Control group**	
**The frequency of drinking days past 3 months**	**Mean/Prob (95% CI)**	**Mean/Prob (95% CI)**	**ARR(95% CI)[e]**	**Mean/Prob (95% CI)**	**Mean/Prob (95% CI)**	**ARR(95% CI)[e]**
Baseline	0.93 (0.26-3.30)	1.28 (0.43-3.81)		0.38 (0.13-1.13)	0.66 (0.27-1.61)	
Follow up	2.46 (0.63-9.58)	1.60 (0.53-4.85)	2.13 (0.55-8.31)	0.98 (0.31-3.15)	0.87 (0.35-2.20)	1.92 (0.38-9.55)
**Any binge drinking[b] during the past 3 months**			**AOR(95% CI)[f]**			**AOR(95% CI)[f]**
Baseline	4.5% (1.1%-16.6%)	9.1% (3.0%-24.5%)		3.3% (0.8%-13.2%)	6.4% (2.0%-18.5%)	
Follow up	7.4% (1.9%-25.2%)	7.0% (2.2%-20.5%)	2.30 (0.56-9.40)	6.0% (1.5%-21.3%)	5.3% (1.6%-15.9%)	2.30 (0.58-9.12)
**The frequency of cannabis use in days past 3 months**			**ARR(95% CI)[g]**			**ARR(95% CI)[g]**
Baseline	3.21 (0.43-23.9)	1.12 (0.20-6.40)		1.29 (0.23-7.36)	0.95 (0.19-4.62)	
Follow up	1.84 (0.22-15.15)	1.33 (0.22-8.07)	0.48 (0.05-4.84)	2.09 (0.30-14.5)	1.17 (0.23-5.98)	1.31 (0.10-17.44)
**The frequency of E-cigarettes use in days past 3 months**			**ARR(95% CI)[h]**			**ARR(95% CI)[h]**
Baseline	4.99 (0.68-36.35)	2.11 (0.34-13.22)		2.50 (0.58-10.76)	2.89 (0.54-15.37)	
Follow up	3.46 (0.34-32.59)	2.76 (0.42-18.35)	0.53 (0.06-4.73)	2.52 (0.47-13.48)	3.75 (0.76-18.47)	0.77 (0.07-9.13)

Abbreviations: Prob, probability; ARR, adjusted rate ratio; AOR, adjusted odds ratio; DID, difference in differences.
[a] Predicted probability is derived from the models.
[b] The criteria for binge drink were defined as follows: females 14-17 years old or males/trans/other genders 12-13 years having 3 drinks containing alcohol on one occasion; females 18 years old or males/other genders 14-15 years old having 4 drinks containing alcohol on one occasion; and males/other genders 16-18 years old having 5 drinks containing alcohol on one occasion[1].
[c] All models adjust for age, gender, parent education, perceived general health, depression and anxiety screening, with the 3way interactions with school types and time point[3].
[d] Difference in differences represents the difference in the change of the outcome over time between the SBI vs the control arm.
[e] Difference in the changes in the rate of drinking days during the past 3months between SBI and control groups from the baseline to the follow-up.
f The estimated odds ratio compares the increases from the baseline to follow-up in binge drinking during the past 3 months between the SBI and the control arms.
[g] Difference in the changes in the rate of cannabis use in days during the past 3months between SBI and control groups from the baseline to the follow-up.
[h] Difference in the changes in the rate of e-cigarette use in days during the past 3months between SBI and control groups from the baseline to the follow-up

© 2022 Levy S et al. *JAMA Network Open*.

eTable 10. Differences in Substance Use Behavior Changes Between SBI and Control School Grades From Baseline to Follow-up When SBI Groups Received the CRAFFT

eTables 10 and 11 show the differences in substance use behavior changes from the baseline to follow-up between SBI and control school-grades, stratified by screening tools.

Screening: CRAFFT	**Weighted, adjusted[b]: Grade 7&8**			**Weighted, adjusted[b]: Grade 9&10**		
	**Marginal Predictive Values[c]**		**DID[d]**	**Marginal Predictive Values[c]**		**DID[d]**
**Variables**	**SBI group**	**Control group**		**SBI group**	**Control group**	
**Drinking days**	**Mean/Prob (95% CI)**	**Mean/Prob (95% CI)**	**ARR (95% CI)[e]**	**Mean/Prob (95% CI)**	**Mean/Prob (95% CI)**	**ARR(95% CI)[e]**
**past 3 months**						
Baseline	0.30 (0.11-0.80)	0.27 (0.10-0.70)		1.27 (0.46-3.54)	1.36 (0.51-3.60)	
Follow up	0.63 (0.24-1.68)	0.50 (0.20-1.25)	1.14 (0.46-2.87)	2.66 (0.91-7.72)	1.55 (0.57-4.20)	1.82 (0.63-5.31)
**Any binge drinking [a]**			**AOR (95% CI)[f]**			**AOR(95% CI)[f]**
**past 3 months**						
Baseline	2.5% (0.7%-8.1%)	1.5% (0.4%-6.2%)		6.0% (2.1%-15.9%)	8.3% (3.0%-20.5%)	
Follow up	3.6% (1.2%-10.6%)	3.0% (0.9%-9.3%)	0.73 (0.14-3.85)	7.1% (2.4%-19.3%)	6.3% (2.2%-16.8%)	1.62 (0.57-4.63)
**Cannabis use**			**ARR (95% CI)[g]**			**ARR(95% CI)[g]**
**days past 3 months**						
Baseline	0.63 (0.17-2.26)	0.28 (0.07-1.10)		3.99 (0.94-17.06)	2.09 (0.51-8.54)	
Follow up	2.26 (0.64-7.99)	3.55 (0.97-13.05)	0.28 (0.06-1.26)	4.33 (0.97-19.31)	2.72 (0.65-11.30)	0.83 (0.13-5.24)
**E-cigarettes use**			**ARR (95% CI)[h]**			**ARR(95% CI)[h]**
**days, past 3 months**						
Baseline	0.68 (0.12-3.71)	0.45 (0.09-2.25)		4.47 (0.80-24.84)	3.16 (0.55-18.02)	
Follow up	1.85 (0.34-10.1)	3.02 (0.59-15.31)	0.41 (0.09-1.92)	4.44 (0.67-29.32)	4.81 (0.82-28.21)	0.65 (0.11-4.00)

Abbreviations: CRAFFT (Car, Relax, Alone, Forget, Friends, Trouble risk screening); Prob, probability; ARR, adjusted rate ratio; AOR, adjusted odds ratio. DID, difference in differences.
[a] The criteria for binge drink were defined as follows: females 14-17 years old or males/trans/other genders 12-13 years having 3 drinks containing alcohol on one occasion; females 18 years old or males/other genders 14-15 years old having 4 drinks containing alcohol on one occasion; and males/other genders 16-18 years old having 5 drinks containing alcohol on one occasion[3].
[a] All models are overlap-weighted and are adjusted for age, gender, parent education, perceived general health, depression and anxiety screening, with the 3way interactions with school types and time point
[b] Predicted probability is derived from the models.
[c] The intervention effect represents the difference in the change of the outcome over time between the SBI vs the control arm.
[d] Difference in the changes in the rate of drinking days during the past 3months between SBI and control groups from the baseline to the follow-up.
[e] The estimated odds ratio comparing the increases from the baseline to follow-up in binge drinking during the past 3 months between the SBI to the control arms.
[f] Difference in the changes in the rate of cannabis use in days during the past 3months between SBI and control groups from the baseline to the follow-up.

© 2022 Levy S et al. *JAMA Network Open*.

eTable 11. Differences in Substance Use Behavior Changes Between SBI and Control School Grades From Baseline to Follow-up When SBI Groups Received the CRAFFT2 and NIAAA

	**Weighted, adjusted[b]: Grade 7&8**		
Screening: CRAFFT2/NIAAA	**Marginal Predictive Values[c]**		**DID[d]**
**Variables**	**SBI group**	**Control group**	
**Drinking days**	**Mean/Prob (95% CI)**	**Mean/Prob (95% CI)**	**ARR(95% CI)[e]**
**past 3 months**			
Baseline	0.53 (0.12-2.41)	0.48 (0.20-1.12)	
Follow up	0.82 (0.19-3.61)	0.66 (0.28-1.54)	1.12 (0.43-2.90)
**Any binge drinking[a]**	**Prob**	**Prob**	**AOR(95% CI)[f]**
**past 3 months**			
Baseline	2.7% (0.6%-11.7%)	1.4% (0.3%-6.2%)	
Follow up	4.2% (1.0%-15.3%)	3.0% (0.9%-9.6%)	0.74 (0.10-5.70)
**Cannabis use**	**Mean**	**Mean**	**ARR(95% CI)[g]**
**days past 3 months**			
Baseline	1.32 (0.16-11.13)	0.52 (0.15-1.79)	
Follow up	2.11 (0.27-16.58)	2.12 (0.59-7.57)	0.39 (0.08-1.90)
**E-cigarettes use**	**Mean**	**Mean**	**ARR(95% CI)[h]**
**days, past 3 months**			
Baseline	1.96 (0.18-20.85)	0.89 (0.24-3.36)	
Follow up	3.61 (0.36-36.05)	2.15 (0.55-8.33)	0.76 (0.17-3.30)

Abbreviations: CRAFFT (Car, Relax, Alone, Forget, Friends, Trouble risk screening); Prob, probability; ARR, adjusted rate ratio; AOR, adjusted odds ratio. DID, difference in differences.; NIAAA, National Institute on Alcohol Abuse and Alcoholism.

[a] The criteria for binge drink were defined as follows: females 14-17 years old or males/trans/other genders 12-13 years having 3 drinks containing alcohol on one occasion; females 18 years old or males/other genders 14-15 years old having 4 drinks containing alcohol on one occasion; and males/other genders 16-18 years old having 5 drinks containing alcohol on one occasion[3].

[b] All models are overlap-weighted and are adjusted for age, gender, parent education, perceived general health, depression and anxiety screening, with the 3way interactions with school types and time point. The evaluation was done among middle school grades as CRAFT2/NIAAA were used among middle schools only.

[c] Predicted probability is derived from the models.

[d] The intervention effect represents the difference in the change of the outcome over time between the SBI vs the control arm.

[e] Difference in the changes in the rate of drinking days during the past 3months between SBI and control groups from the baseline to the follow-up.

[f] The estimated odds ratio comparing the increases from the baseline to follow-up in binge drinking during the past 3 months between the SBI to the control arms.

[g] Difference in the changes in the rate of cannabis use in days during the past 3months between SBI and control groups from the baseline to the follow-up.

[h] Difference in the changes in the rate of e-cigarette use in days during the past 3months between SBI and control groups from the baseline to the follow-up.

© 2022 Levy S et al. *JAMA Network Open*.

eFigure. Mirrored Density Plot of Propensity Scores in Full Baseline School Grades, Including 13 School Grades That Received SBI and 21 Control School Grades, Before and After Overlap Weighting[a]

eFigure shows the propensity score distributions before and after the application of the weights for the full baseline cohort.

**Unweighted**	**Weighted**
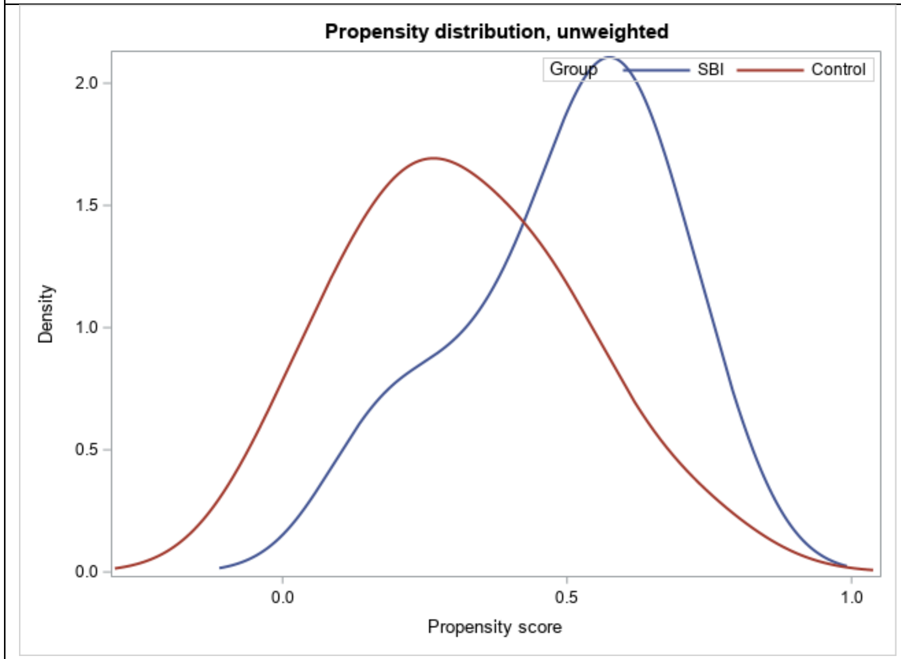	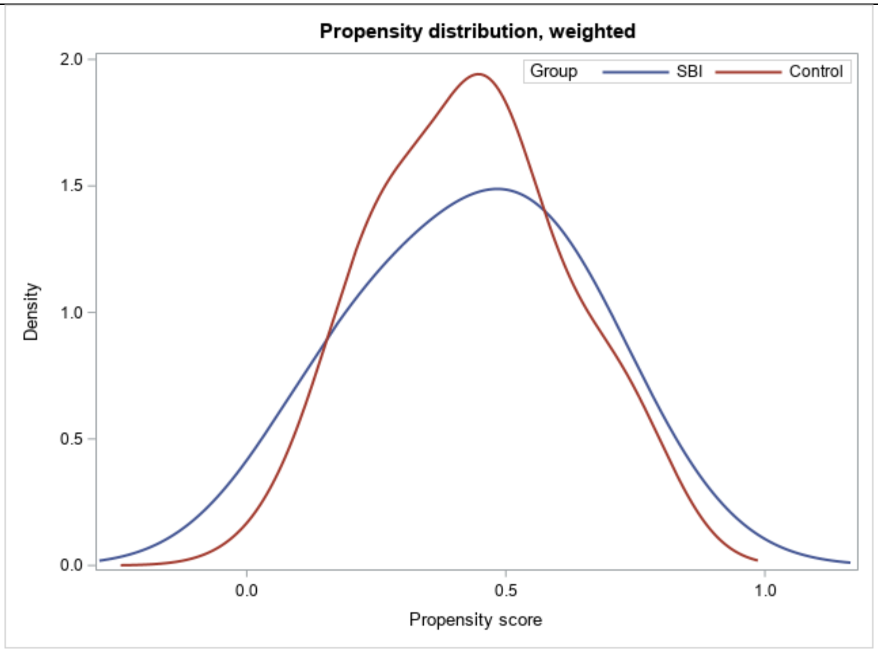

[a] Propensity scores were derived from propensity score models that included ecological and demographic including per annual per-pupil expenditure, the proportion of pupils with high need, economic disadvantages, the proportion of pupils with grade7, grade10, female gender, white race, and parental education, perceived negative parental attitudes towards teen substance use, and perceived positive family history of alcohol problems).

© 2022 Levy S et al. *JAMA Network Open*.

eReferences

1. Arroll B, Goodyear-Smith F, Crengle S, et al. Validation of PHQ-2 and PHQ-9 to screen for major depression in the primary care population. Ann Fam Med. 2010;8(4):348-353. doi:10.1370/afm.1139
2. Skapinakis P. The 2-item Generalized Anxiety Disorder scale had high sensitivity and specificity for detecting GAD in primary care. Evid Based Med. 2007;12(5):149. doi:10.1136/ebm.12.5.149
3. Donovan JE. Estimated blood alcohol concentrations for child and adolescent drinking and their implications for screening instruments. Pediatrics. 2009;123(6). doi:10.1542/peds.2008-0027

© 2022 Levy S et al. *JAMA Network Open*.
